# Porphyrin-Embedded Silicate Materials for Detection of Hydrocarbon Solvents

**DOI:** 10.3390/s110100886

**Published:** 2011-01-14

**Authors:** Brandy J. Johnson, Nicole E. Anderson, Paul T. Charles, Anthony P. Malanoski, Brian J. Melde, Mansoor Nasir, Jeffrey R. Deschamps

**Affiliations:** Center for Bio/Molecular Science and Engineering, Naval Research Laboratory, Washington, DC 20375, USA; E-Mails: nanders2@umd.edu (N.E.A); paul.charles@nrl.navy.mil (P.T.C.); anthony.malanoski@nrl.navy.mil (A.P.M.); brian.melde@nrl.navy.mil (B.J.M.); mansoor.nasir@nrl.navy.mil (M.N.); jeff.deschamps@nrl.navy.mil (J.R.D.)

**Keywords:** mesoporous, organosilica, fluorescence, porphyrin, hierarchical, detection, solvent

## Abstract

The development of porphyrin-embedded mesoporous organosilicate materials for application to the detection of volatile hydrocarbon solvents is described. Design of the receptor and optical indicator construct begins with parallel selection of the porphyrin indicator and design of the mesoporous sorbent. For the porphyrin indicator, high binding affinity and strong changes in spectrophotometric character upon target interaction are desired. The sorbent should provide high target binding capacity and rapid binding kinetics. A number of porphyrin/metalloporphyrin variants and organosilicate sorbents were evaluated to determine the characteristics of their interaction with the targets, benzene, toluene, and hexane. The selected porphyrin candidates were covalently immobilized within a benzene-bridged sorbent. This construct was applied to the detection of targets using both fluorescence- and reflectance-based protocols. The use of red, green, and blue (RGB) color values from the constructs in a highly simplified detection scheme is described.

## 1. Introduction

Mesoporous silicates have been widely described in sensing applications in recent years. They are typically applied as receptors or as scaffolds for the immobilization of receptors and reporters. Porphyrins are also widely reported in sensing applications in which their binding and/or spectrophotometric characteristics are utilized. The work described here combines these two materials in a single construct designed to provide enhanced performance through utilization of combined characteristics. Specifically, the silicate component is used to provide stability to the porphyrin component as well as target binding affinity and a large surface area for indicator immobilization. The porphyrin also provides binding affinity and acts as a transducer for the construct.

The targets of interest here are those often associated with contamination in areas near petroleum processing and handling sites. BTEX (benzene, toluene, ethylbenzene, xylene) are naturally occurring in petroleum products. The individual compounds are also used in a range of manufacturing and production processes. The BTEX compounds are volatile, aromatic hydrocarbons. They are clear, colorless, and flammable. Exposure to high levels of BTEX is associated with skin and sensory irritation, respiratory distress, and depression of the central nervous system. Prolonged exposure to BTEX has been associated with liver and kidney dysfunction and benzene is a known carcinogen. In addition to evaporation into the environment, these compounds can dissolve into water sources or adsorb to soils presenting greater health hazards as a result of increased concentrations.

**Figure 1.**
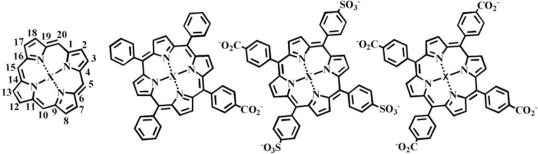
Structures of the porphyrin parent compound and those porphyrins used in the presented studies; from left to right: porphine; 5-mono(4-carboxyphenyl)-10, 15,20-triphenyl porphine (C_1_TPP); meso-tri(4-sulfonatophenyl)mono(4-carboxyphenyl) porphine (C_1_S_3_TPP); and meso-tetra(4-carboxyphenyl) porphine (C_4_TPP). Metal complex formation occurs through interaction of the central nitrogen atoms indicated here with an ‘X’.

Porphyrins and phthalocyanines have been applied to the detection of volatile, aromatic hydrocarbons previously [1–8]. Strong π-π*-interactions between the indicator macrocycle (Figure 1) and the target provide the potential for large changes in the spectrophotometric characteristics of the porphyrin (or phthalocyanine). These interactions also provide binding affinity between the target and indicator making it possible to utilize the porphyrin as a receptor, an indicator, or both. Most applications employ the porphyrin in both capacities. Binding and spectrophotometric characteristics can be altered through modification to the porphyrin structure or incorporation of a metal into the central coordination site. Porphyrins are often incorporated into thin films for these sensing applications [1–3,7,8] which may rely on a number of differing detection techniques including optical and electrochemical.

**Figure 2.**
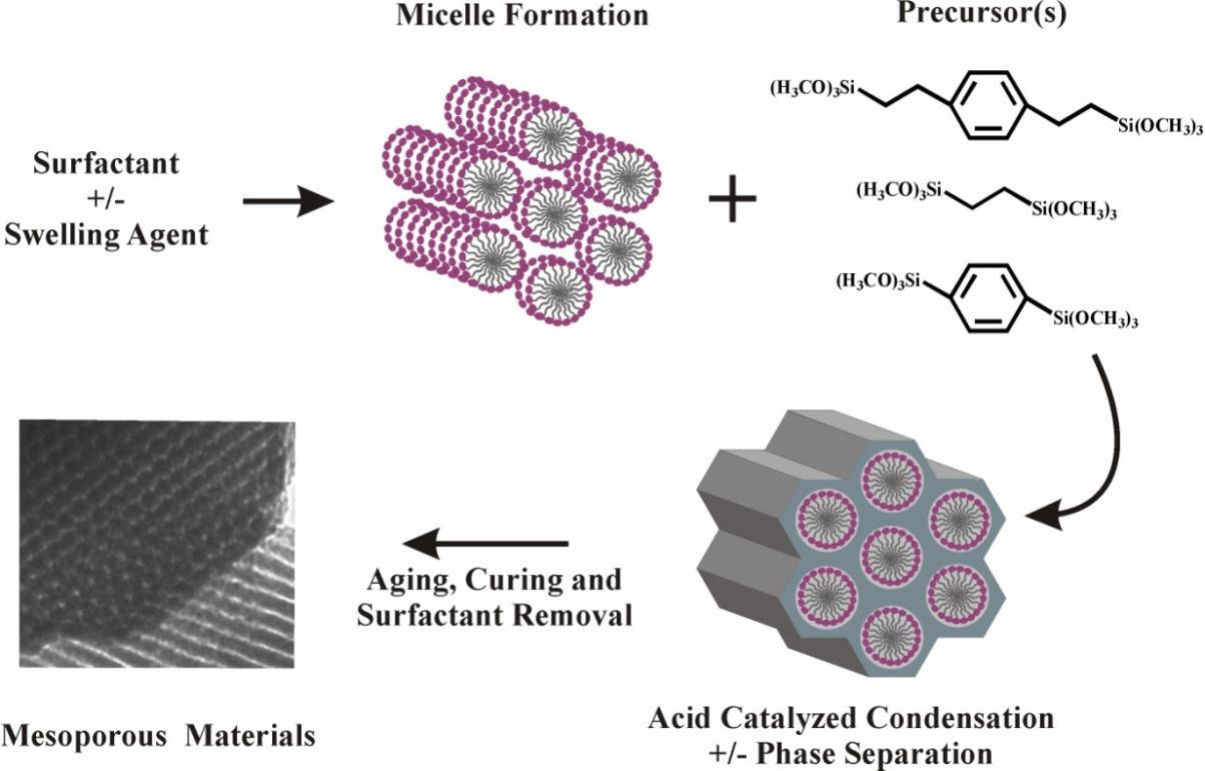
Synthesis of organosilicate sorbents.

Porous silicate materials offer high surface areas, open pore networks, and tunable binding characteristics to sensing applications. Detection techniques including optical absorption spectroscopy, quartz crystal microbalance (QCM), and FTIR have been utilized for aromatic hydrocarbon sensing applications based on these materials [4,9–18]. We have previously reported on the potential of mesoporous organosilicate materials for the semi-selective adsorption of targets [19,20]. The organosilicates offer average pore diameters ranging from 2 to 10 nm. This pore size can be controlled in order to limit the admission of analytes into the pore volume. Further control of target interactions is possible through selection of the precursors which comprise the pore walls, inclusion of an imprint template as part of the surfactant mixture [19], or through post-synthesis grafting of functional moieties to the pore walls (Figure 2).

The study described here details the selection of porphyrin and organosilicate components in the design of a construct applicable to the optical detection of hydrocarbon solvents. Benzene, toluene, and hexane were selected as targets for the study in order to address both aromatic, *i.e.*, BTEX components, and non-aromatic hydrocarbons. The construct is applied to fluorescence based detection of the targets. Data generated using an alternative detection method utilizing RGB (red green blue) color values from reflectance images is also presented.

## 2. Results and Discussion

The targets of interest to this study are hydrocarbons such as toluene, benzene, and hexanes. As we have described previously [21], the design of a porphyrin-embedded material (PEM) directed at the detection of a specific target begins with the selection of the organic bridging groups that will provide affinity and design of an imprint template based on a target analog. In the case of this effort, we are seeking to detect a range of compounds rather than a single compound. As a result, the sorbent scaffold will not utilize an imprint template. Instead, the sorbent needs to offer binding capacity for the range of compounds of interest. This broad binding requirement necessitates a compromise in the precursors used to provide affinity and capacity for all targets rather than high affinity for a single target. Once a sorbent has been designed, surface modification is undertaken to provide sites for porphyrin incorporation. The porphyrin component is selected based on porphyrin-target binding affinity and the resultant changes in the spectrophotometric characteristics of the porphyrin. Optimization of sorbent and porphyrin components is completed in parallel.

### 2.1. Organosilicate Sorbent

A number of organosilicate materials were synthesized and evaluated for their potential to adsorb volatile organic compounds (VOCs) such as those comprising BTEX (mixture of benzene, toluene, ethylbenzene, xylene). Aromatic BTEX components possess a mechanism for interaction with the sorbent via π-bond interactions. Phenyl rings within the organosilicates provided by precursors such as 1,4-bis(triethoxysilyl)benzene or phenyltriethoxysilane offer potential sites for interaction with these targets. Our approach to adsorption of non-aromatic hydrocarbons was to attempt to provide a favorable environment based on hydrophobicity. The materials produced are listed in Table 1 along with their characteristics as determined by nitrogen porosimetry.

**Figure 3.**
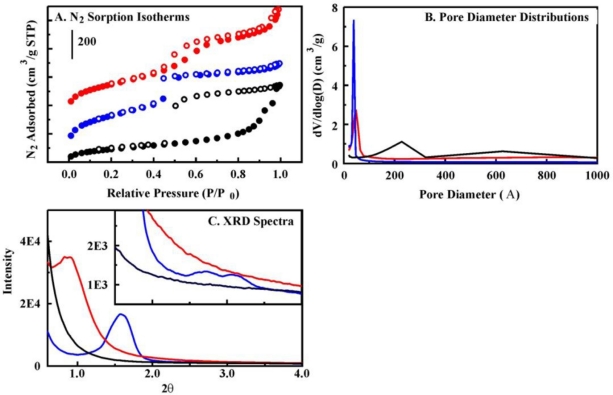
Structural characterization. Panel A, nitrogen sorption isotherms (BP2 offset by 325 cm^3^/g). Panel B, pore size distributions. Panel C, XRD spectra. B100 (blue), BP2 (red), OTS (black).

**Figure 4.**
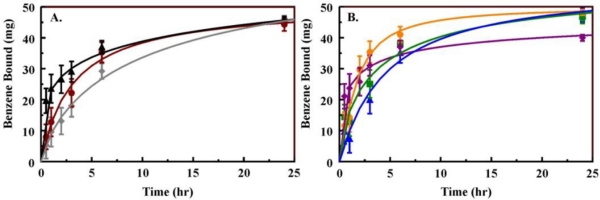
Binding kinetics from vapor phase. Shown here are the kinetics of benzene binding for each of the organosilicate materials. Benzene (53 mg) was allowed to diffuse within a volume of 125 mL for the indicated time (20 °C). Panel A, B100 (black), OTS (red), and BP2 (gray). Panel B, DEB (orange), Ph1 (green), PhE1 (blue), and TM1 (purple).

The materials utilized in these studies were diverse, offering a range of chemical structures and materials morphology (Figure 3 and Supporting Information, Figure S1). The targets of this effort are highly volatile with vapor pressures ranging from 1 to 20 kPa (20 °C). Physisorption of these types of targets is a complex process involving a number of forces and interactions of the hydrocarbon molecules with the pore surfaces. The different materials synthesized offer variations on the typical forces involved in physisorption (hydrogen bonding, dipole-dipole interactions, induced dipole, etc). These variations are provided both through the chemical groups and through the flexibility of those groups within the materials structure. Pore curvature, accessibility, and interconnectivity further alter the potential target-sorbent interactions. As shown in Table 1 and Figure 4, the interaction of benzene with the sorbents demonstrates that the materials offer varying capacities and interaction kinetics. For example, TM1 offers more rapid kinetics than BP2, but the binding capacity of BP2 is higher than that of TM1 both on per gram and per surface area bases (200 mg/g *vs.* 180 mg/g and 310 μg/m^2^ *vs.* 240 μg/m^2^). TM1 is rigid with high site homogeneity (silicate framework with pendant methyl groups) and provides ease of diffusion through large diameter pores leading to the more rapid kinetics (Figure 4). BP2 offers sites of higher affinity, but, due to varying orientation of the biphenyl groups in the pore walls, the specific affinity of the sites will vary. In addition, the pore diameter is smaller. This greater curvature both offers more surface area for interaction with a given molecule and restricts the rate of diffusion throughout the material.

A similar balance in characteristics can be noted for the other materials considered. The large hysteresis in the nitrogen sorption isotherm for OTS and its broad pore size distribution indicate complexity in the pore structure of that material related to pore shape and/or interconnectivity [22]. Lack of mesoscale order in OTS was also confirmed by the absence of reflections in its powder XRD pattern (Figure 3). PhE1 has the narrowest pore diameter and the slowest adsorption kinetics. Having considered this balance in each of the materials, B100 was selected as the sorbent for porphyrin incorporation. The material offers well organized pores with hexagonal packing, evidenced by observation of (100), (110), and (200) XRD reflections, as well as high surface area, rapid binding kinetics, and high total target capacity. B100 was also found to offer a higher total BTEX capacity than the other sorbents evaluated (Supporting Information, Figure S2).

### 2.2. Porphyrin Indicator

**Figure 5.**
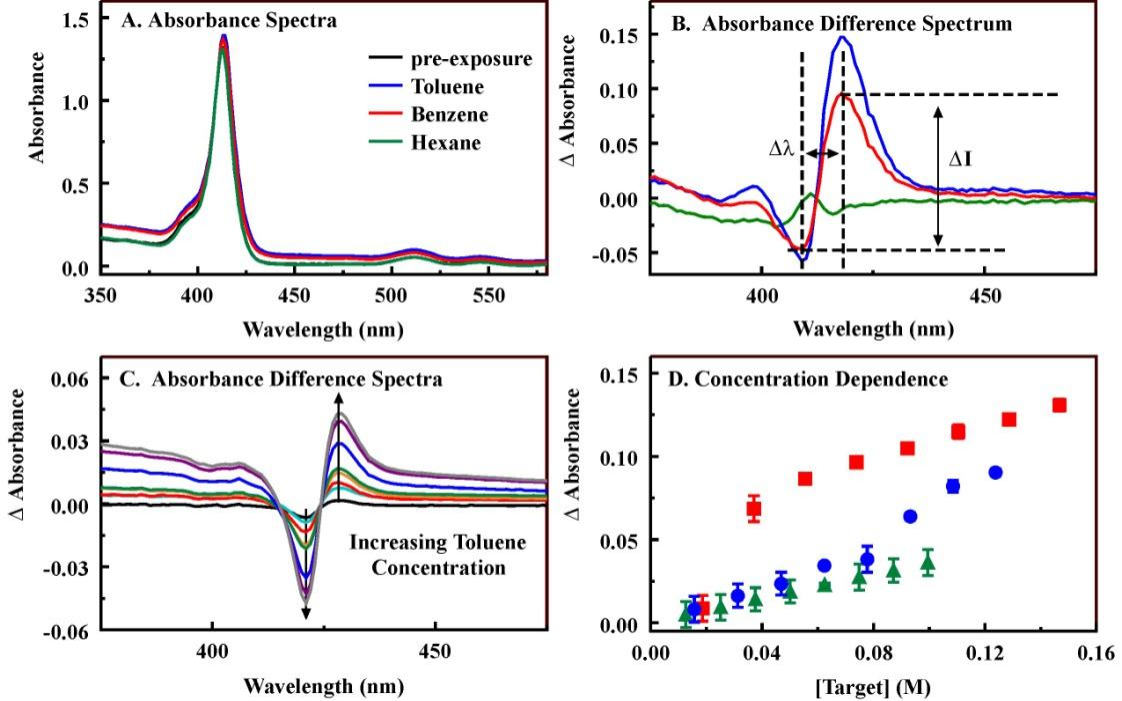
Changes in the porphyrin absorbance characteristics upon interaction with targets. Panel A, absorbance spectra of MnC_1_TPP (4 μM) in the absence (black) and presence of benzene (red), toluene (blue), and hexanes (green) (200 mM, in 95% methanol). Panel B, difference spectra calculated as post-exposure minus pre-exposure absorbance from Panel A. In this spectrum, the distance between the peak position and the trough position (Δλ) and the difference between the peak height and the trough depth (ΔI) are indicated for the benzene interaction. Panel C, difference spectra resulting from the exposure of ZnC_4_TPP (3.3 μM) to varying concentrations of toluene. Panel D, concentration dependence of the interaction between ZnC_4_TPP and the three targets. Results for additional metalloporphyrins are provided in Supporting Information, Figure S3.

Three porphyrin structures (Figure 1) were combined with 20 metal salts to generate 63 porphyrin and metalloporphyrin variants (Supporting Information, Table S1). The direct interaction of these variants with the targets, hexane, benzene, and toluene, was investigated using a rapid screening microplate format in order to select those porphyrins which offered the highest binding affinities and largest changes in spectrophotometric characteristics upon binding [21]. A single porphyrin concentration was challenged using a single target concentration. The interaction of porphyrin and target results in a change in the π-bond conformation of the porphyrin yielding a change in the spectrophotometric characteristics of the porphyrin. These changes can include increases and decreases in absorbance intensity at one or many wavelengths. In order to simplify analysis of these changes, we have utilized an absorbance difference spectrum that is calculated as the point-by-point subtraction of the pre-exposure spectrum from the post-exposure spectrum. Figure 5 presents examples of pre- and post-exposure spectra and difference spectra. Here, MnC_1_TPP (4 μM) was exposed to target (200 mM in methanol). In the difference spectra, both the separation between the peak and the trough (Δλ) and the total change in intensity (peak intensity minus the trough intensity, ΔI) provide an indication of the binding affinity between the target and the porphyrin. This binding affinity is directly related to the potential for application of the porphyrin as an indicator (sensitivity) [23]. Table 2 provides examples of Δλ and ΔI values obtained for some of the target-porphyrin pairs (Additional data is provided in Supporting Information, Table S2). Using this technique, several candidates are typically selected based on those pairs showing the largest Δλ or ΔI values. The wavelength shifts for the porphyrins ranged between 8 and 12 nm. Several porphyrin-target interactions also lead to a decrease in intensity at one wavelength with no accompanying increase (MnC_4_TPP, for example). Because of this narrow range, Δλ values were not considered in the selection of candidates. Overall, the C_1_TPP variants showed higher changes in intensity than the other porphyrins with FeC_1_TPP demonstrating the most significant changes. Because we wanted to characterize the performance of different porphyrin structures for further validation of the rapid screening techniques used, we included the metalloporphyrins providing the three largest intensity changes as well as C_1_TPP for additional characterization. We also selected four C_4_TPP variants including the base porphyrin and the three metalloporphyrins providing the largest changes in intensity. C_1_S_3_TPP was not characterized further due to the very small intensity changes noted for that porphyrin and the metalloporphyrin variants upon exposure to the targets (Supporting Information, Table S2).

Based on the results of the microplate screening technique, several porphyrins were further evaluated through the generation of a binding isotherm (Figure 5 and Supporting Information, Figure S3 and Table S3). The change in intensity for a given target-porphyrin interaction is dependent on the concentration of both the target and the porphyrin. Here, the porphyrin concentration was fixed and the concentration of the targets was varied. Using the changes in intensity (peak or trough) as a function of the target concentration, the Benesi-Hildebrand method can be used to obtain binding constants (K_11_) and changes in extinction coefficients resulting from porphyrin-target complex formation (Δ′_11_) (Table 2 and Supporting Information, Table S3) [21,24,25]. This data set provides a more complete picture for the expected performance of a given porphyrin in a sensing application. Relative performance for the various porphyrin-target combinations can also be evaluated. Using these screening techniques, iron and manganese complexes of C_1_TPP and C_4_TPP were selected for incorporation into the sorbent material.

### 2.3. Fluorescence-Based Detection

**Figure 6.**
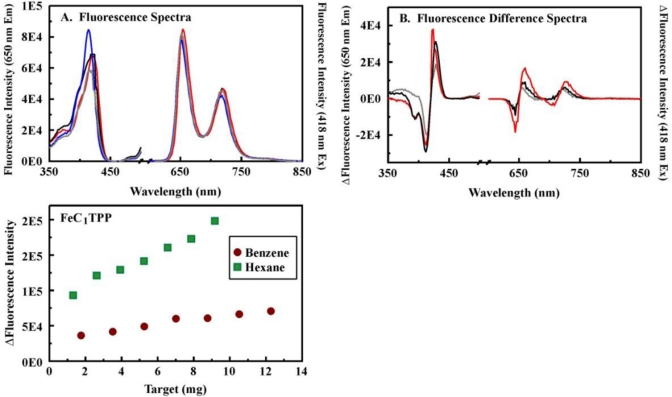
Interaction of targets with FeC_1_TPP-embedded B100. Panel A, fluorescence excitation and emission spectra for the FeC_1_TPP-embedded B100 material (2 mg) in the absence (blue) and presence of 3.5 mg (gray), 5.3 mg (black), and 12 mg (red) benzene. Panel B, difference fluorescence spectra for exposure of the material to benzene. Panel C, binding isotherms for the interaction of FeC_1_TPP-embedded B100 with benzene (425 and 414 nm) and hexanes (417 and 415 nm) based on the peak/trough difference in intensity. Results for additional metalloporphyrins are provided in Supporting Information, Figures S4 through S9.

Porphyrins (C_1_TPP and C_4_TPP) were covalently incorporated into the organosilicate scaffold (B100), and metalloporphyrin variants were generated by refluxing the porphyrin-embedded material in a solution of the appropriate metal. Porphyrin loading levels which provide sensitive detection and do not interfere with the function of the sorbent are desirable. Here, porphyrin loading levels are controlled through regulation of the degree of amine-functionality on the surface of the materials. We have previously presented data showing the impact of loading levels on the spectrophotometric characteristics of the porphyrins and on their response to target challenge [21]. Loading levels here were selected based on those studies. The fluorescence response of the porphyrin-embedded materials was evaluated in a microplate format using a fixed mass of dry sorbent with a fixed total volume of target added (target + methanol). Control spectra were collected using methanol. Pre- and post-exposure fluorescence spectra were used to calculate difference spectra, and peak/trough intensity differences were used for comparison of the materials (Figure 6, Table 3, and Supporting Information, Figures S4 through S9).

Porphyrins showing high binding affinity and/or large changes in absorbance upon interaction with targets in solution were expected to provide the most effective indicators when immobilized within the sorbent materials. MnC_1_TPP, for example, showed a high binding affinity and moderate changes in absorbance upon interaction with hexane in solution (Supporting Information, Table S3 and Figure S3). The MnC_1_TPP-embedded B100 material showed large changes in fluorescence intensity and saturation of the indicator was reached at <3.5 g/g indicating relatively high target uptake into the sorbent (Supporting Information, Figure S6). A similar pattern was maintained for the other C_1_TPP variants (Supporting Information, Figures S4, S5, and S6). C_4_TPP indicators did not perform as expected (Supporting Information, Figures S7 and S8). MnC_4_TPP, for example, demonstrated moderate binding affinities and changes in absorbance upon exposure to targets in solution (Supporting Information, Table S3). No changes in fluorescence were observed upon interaction of MnC_4_TPP-embedded B100 with the targets. C_4_TPP, for which large changes in absorbance were observed in solution (Table 2), demonstrated changes in fluorescence only upon interaction with toluene (Supporting Information, Figure S7). It is possible that the multiple carboxylic acid groups on this porphyrin lead to co-facial interaction between it and the pore walls resulting in reduced target sensitivity for the porphyrin (due to π-bond perturbation) and/or reduced target affinity by the sorbent (porphyrin occupation of binding sites).

### 2.4. Alternative Detection Techniques

Having demonstrated the performance of the PEM materials using a commercially available instrument, our focus shifted to detection methods that could be more readily adapted to portable formats. Fluorescence-based detection utilizing spectra (as opposed to single wavelengths) as described above tends to have power and resolution demands that preclude using the technique in highly miniaturized formats. The use of single or pairs of wavelengths for porphyrin-based detection has been proposed [26–28]. While this approach is useful for systems focusing on the detection of a single target and, therefore, a single set of characteristic interaction wavelengths, the approach does not allow for detection of classes of molecules. As seen above, the benzene-FeC_4_TPP interaction produces a peak-trough pair at 648 and 663 nm while the toluene-FeC_4_TPP interaction produces a peak-trough pair at 664 and 670 nm. A method that provides interrogation of a wider range of wavelengths is desirable. A previous effort reported the use of a flatbed scanner for monitoring changes in porphyrin characteristics upon exposure to solvents [2,4,8]. While this effort demonstrated that visually discernable color changes occurred upon target exposure, methods were not proposed for utilizing the technique, and sensitivity to varying concentrations was not demonstrated.

**Figure 7.**
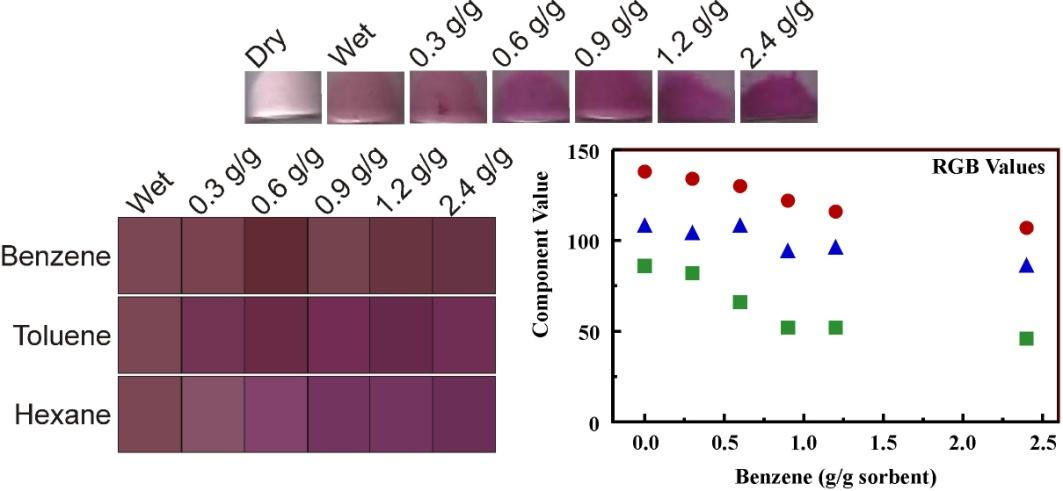
Interaction of FeC_1_TPP-embedded B100 with targets. Shown here are scanner images of the material following exposure to varying concentrations of benzene. Also shown are simulated images generated based on average RGB values for FeC_1_TPP-embedded B100 following exposure to varying target concentrations and the dependence of RGB image color values on target concentration for the interaction of benzene with FeC1TPP-embedded B100 (red circles = R, blue triangles = B, green squares = G). Complete data for other materials/targets is presented in the Supporting Information, Figures S4 through S9.

Figure 7 presents representative data utilizing FeC_1_TPP-embedded B100 for the detection of benzene, toluene, and hexane at varied concentrations. Scanner images were collected using 5 mg of the porphyrin-embedded sorbents in clear shell vials. Images were collected of the dry sorbents and sorbents exposed to 25 μL methanol as controls. The total target volume for all samples was 25 μL with the balance being methanol. The change in the reflectance for the FeC_1_TPP-embedded B100 as the benzene concentration varies is visually discernable. The average RGB values for the scanned images were collected and used to generate simulated images (Figure 7). These values were also plotted *vs.* the concentration of target applied. As shown in the figure, exposure of the FeC_1_TPP-embedded B100 material to benzene resulted in significant changes in the R and G values with little variation in the B value of the image. Several materials, selected based on observation of changes in their spectrophotometric characteristics upon target challenge (Sections 2.2 and 2.3), were evaluated in this way. Complete results are presented in the Supporting Information, Figures S11 through S16. Hexanes did not produce strong changes in the reflectance of any of the materials except FeC_1_TPP embedded B100. This result was expected based on the fluorescence studies presented above (Section 2.3). On benzene or toluene challenge, strong reflectance changes were noted for MnC_4_TPP-, C_1_TPP- and FeC_1_TPP-embedded B100.

**Figure 8.**
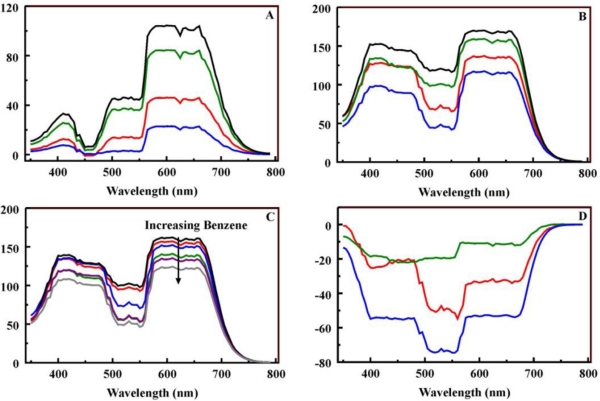
Reflectance spectra. Shown here are the reflectance spectra generated for MnC_4_TPP-embedded B100 (black, Panel A) and C_1_TPP-embedded B100 (black, Panel B) from the RGB values using the algorithm. Also shown are the spectra observed following exposure (2.4 g/g) of the materials to benzene (red), toluene (blue), and hexane (green). Panel C shows the change in the reflectance spectrum for FeC_1_TPP-embedded B100 as the concentration of benzene is increased from 0 to 2.4 g/g. Panel D presents difference spectra to highlight the variations specific to each target (benzene (red), toluene (blue), and hexane (green)) on interaction with C_1_TPP-embedded B100. Complete reflectance spectra sets for other materials/targets are presented in the Supporting Information, Figures S11 to S16.

The question that we sought to address with this data set was whether there was a robust method using reflectance RGB values alone that provided enough information for discrimination of targets and target concentrations using the porphyrin embedded organosilicate materials. A transformation from RGB values to some type of spectra is more robust because the contributions from each value in the RGB set are combined. Other groups have studied the conversion of spectra to RGB values and vice versa [29–31]. Here, we have adopted an approach that is intended to produce physically plausible reflectance spectra based on the RGB values taken from the scanned images to allow comparisons that use the information contained in all three RGB values [32]. The method uses RGB weighted sums of seven spectra: white, red, blue, green, cyan, magenta, and yellow (Supporting Information, Figure S10). Figure 8 shows the spectra generated for MnC_4_TPP- and C_1_TPP-embedded B100 before and after exposure to benzene, toluene, and hexane (additional spectra provided in Figures S11 to S16). While the changes are more apparent in the difference spectra (as seen in Section 2.2), both target and concentration dependent characteristics are clear. The processing of these RGB values utilized reflectance spectra at 5 nm resolution to cover the region from 360 to 800 nm. The fluorescence spectra presented above were collected at 1 nm resolution.

## 3. Experimental Section

Meso-tetra(4-carboxyphenyl) porphine (C_4_TPP); 5-mono(4-carboxyphenyl)-10,15,20-triphenyl porphine (C_1_TPP); and meso-tri(4-sulfonatophenyl)mono(4-carboxyphenyl) porphine (C_1_S_3_TPP) were obtained from Frontier Scientific, Logan, UT. 1-Ethyl-3-[3-dimethylaminopropyl]carbodiimide (EDC) was purchased from Pierce Chemical Company (Rockford, IL). 1,4-bis(triethoxysilyl)benzene 96%, 4,4’-bis(triethoxysilyl)biphenyl 95%, 1,2-bis(triethoxysilyl)ethane 96% (BTE), 3-aminopropyltriethoxy silane (APS), tetramethyl orthosilicate (TMOS) 98%, mesitylene 98%, Brij®76, nitric acid 70% (HNO_3_), and *n*-butanol were obtained from Sigma-Aldrich. Methyltriethoxysilane (MTES), bis(trimethoxysilylethyl)benzene (DEB), phenyltriethoxysilane, phenethyltrimethoxysilane, and *n*-octyltrimethoxysilane were purchased from Gelest, Inc., Ethanol (200 proof) from the Warner-Graham Company, and concentrated hydrochloric acid 37% (HCl) from Fisher Scientific, Inc. Pluronic P123 was a generous donation from BASF. Water was deionized to 18.2 MΩ cm using a Millipore Milli Q UV-Plus water purification system.

### 3.1. Materials Synthesis and Morphological Characterization

*Benzene-bridged PMO (B100):* This synthesis followed the procedure of Wang *et al.* [33] in which 2.0 g of Brij 76 was dissolved in 10.0 g of water and 50.0 g of 2 M HCl with stirring at 50 °C. 1,4-bis(triethoxysilyl)benzene (4.225 g) was added and the mixture stirred at 50 °C for 20 h. The mixture was then heated under static conditions for 20 h at 95 °C. White precipitate was collected by vacuum filtration and rinsed with water. Surfactant was removed by refluxing two times for at least 12 h in 1 M HCl in ethanol. Material was collected again by vacuum filtration, washed with ethanol, then water, and dried at 110 °C.

*Biphenyl-bridged PMO (BP2):* This synthesis followed the procedure of Yang *et al.* [34] in which 1.0 g of Pluronic P123 was dissolved in 36.0 g of water and 1.96 g of concentrated HCl with stirring at 35 °C. *n*-Butanol (1.48 g) was added and the solution was stirred 1 h at 35 °C. 4,4’-Bis(triethoxysilyl)biphenyl precursor (3.20 g) was added and the mixture was stirred for 1 d at 35 °C followed by heating under static conditions for 5 d at 95 °C. White precipitate was collected by vacuum filtration and rinsed with water. Surfactant was removed by refluxing twice for at least 12 h in 1 M HCl in ethanol. Material was collected again by vacuum filtration, washed with ethanol, then water, and dried at 110 °C.

*Diethylbenzene-bridged PMO (DEB):* DEB was synthesized as described previously [35]. Briefly, Pluronic P123 (1.9 g) and mesitylene (0.7 g) were dissolved in 7.5 g 0.1 M HNO_3_ with stirring at 60 °C. The mixture was allowed to cool to room temperature prior to drop-wise addition of 2.94 g bis(trimethoxysilylethyl)benzene and stirred until homogeneous. The sol was transferred to a culture tube, sealed, and heated at 60 °C over night (∼18 h) during which a gel formed. The tube was unsealed, heated at 60 °C for 2 d, and then heated at 80 °C for 2 d. Surfactant was extracted by refluxing three times for at least 12 h in 1 M HCl in ethanol, breaking the monolithic material down to a powder. It was collected by vacuum filtration, washed with ethanol, then water, and dried at 110 °C.

*Methyl-silicate mesoporous material (TM1):* This synthesis was adapted from a procedure described by Nakanishi *et al.* [36], substituting 10 mol % of the tetramethyl orthosilicate originally used with methyltriethoxysilane. Pluronic P123 (0.7 g) was dissolved in 16.8 g of 1.0 M HNO_3_ with stirring at 60 °C. The solution was allowed to cool to RT prior to a drop-wise addition of a mixture of 0.927 g TMOS and 0.121 g MTES and stirred until homogeneous. The sol was transferred to a culture tube, sealed, and heated at 60 °C over night (∼18 h) during which a gel formed. The tube was unsealed and heated at 60 °C for 1 d and heated at 80 °C for 7 d. Surfactant was extracted by refluxing two times for at least 12 h in 1 M HCl in ethanol, breaking the monolithic material down to a powder. It was collected by vacuum filtration, washed with ethanol, then water, and dried at 110 °C.

*Phenyl- and alkyl-functionalized ethane-bridged organosilicas:* Syntheses for these materials adapted from a procedure reported by Burleigh *et al.* [37]. Briefly, 4.0 g of Brij 76 was dissolved in 13.1 mL concentrated HCl and 286.9 mL H_2_O with stirring at 50 °C. A silane mixture containing 0.0562 mol Si, 75 mol% Si from 1, 2-bis(triethoxysilyl)ethane and 25 mol% Si from phenyltriethoxysilane (Ph1), phenethyltrimethoxysilane (PhE1), or *n*-octyltrimethoxysilane (OTS), was added drop-wise. The mixture was stirred and heated at 50 °C overnight (≥12 h) and heated under static conditions at 90 °C for 24 h. White precipitate was collected by vacuum filtration and rinsed with water. Surfactant was removed by refluxing three times for at least 12 h in 1 M HCl in ethanol. Material was collected again by vacuum filtration, washed with ethanol, then water, and dried at 110 °C.

N_2_ sorption experiments were completed using a Micromeritics ASAP 2010 at 77 K. Samples were degassed to 1 μm Hg at 100 °C prior to analysis. Surface area was determined by use of the Brunauer-Emmett-Teller (BET) method; pore size was calculated by the Barrett-Joyner-Halenda (BJH) method from the adsorption branch of the isotherm; total pore volume was calculated by the single point method at relative pressure (P/P_0_) 0.97. Powder x-ray diffraction patterns were collected at room temperature using CuKα radiation from a Brüker MICROSTAR-H x-ray generator operated at 40 kV and 20 mA equipped with a 5 mRadian collimator, and a Brüker Platinum-135 CCD area detector. A custom fabricated beamstop was mounted on the detector to allow data collection to approximately 0.4° 2θ (approximately 210 Å) with a sample to detector distance of 30 cm. After unwarping the images, the XRD^2^ plug-in was used to integrate the diffraction patterns from 0.3° to 8.4° 2θ.

Metalloporphyrin variants were prepared by dissolving porphyrin and metal salts in water at a 1:1 molar ratio (refer to Supporting Information, Table S1 for a list of metal salts used). The solutions were heated at 90 °C until dry and the resulting metalloporphyrins were resuspended to a final concentration of 2 mM in deionized water. Porphyrin incorporation into the sorbents was accomplished using amine surface functionalization together with EDC chemistry. Post-synthetic grafting of APS into B100 was accomplished as described previously [21]. Briefly, sorbent material (1.0 g) was added to a solution of APS (1 mL) in toluene (200 mL). The mixture was refluxed overnight before collection by vacuum filtration, rinsing with toluene and ethanol, and drying at 110 °C. Functionalized silicate material (1 g) was placed in a solution of 5 mM EDC and 0.6 mM porphyrin in 100 mM MES buffer (2-(*N*-morpholino)ethanesulfonic acid). The solution was incubated with agitation overnight and rinsed with ethanol and water. Metals were incorporated into porphyrin-functionalized materials by refluxing the sorbent in a solution containing 1 mM metal salt in deionized water overnight.

### 3.2. Target Adsorption and Detection Experiments

For vapor phase adsorption experiments using the organosilicate materials, headspace sampling vials (125 mL; Entech Instruments, Inc) equipped with septum-type sampling ports were employed. Experiments were completed by placing a volume of liquid target in a small tube into the vial with a tube containing sorbent. Vials were then incubated at 20 °C (controlled temperature water bath). Controls were completed in the same manner in the absence of sorbent. Headspace samples were removed from the vials and injected directly for analysis using a gas-tight syringe (Hamilton) in triplicate for analysis. Analysis was performed using an Agilent 6890 Gas Chromatograph (GC) equipped with an Optima-210 (50 m × 0.25 mm × 0.25 μm film) fused silica capillary column (Macherey-Nagel). GC instrument parameters were set for split mode operation with a helium carrier gas (89 mL/min total flow). Pressure was set at 60 kPa with a programmable temperature gradient set initially at 50 °C (hold for 3 min), ramp temperature 8 °C/min to 190 °C (hold 10 min). Detection was accomplished with an FID detector set at 260 °C.

Concentrations for analyte adsorption experiments are provided in the text and figure captions. Prior to HPLC analysis, all solutions were filtered through 0.2 μm PTFE syringe filters to remove the adsorbent and the captured target. The remaining solution was then analyzed and the amount adsorbed was calculated via difference method. Standard curves were generated for each set of experiments to verify sample concentrations and proper instrument function. The HPLC method was adapted from Zoccolillo, *et al.* [38] and employed a Hewlett-Packard 1100 DAD High Performance Liquid Chromatography (HPLC). The stationary phase was a C18 pyramid column (Nucleodur, 250 mm × 4 mm, 5 μm diameter; Sorbent Technologies, Atlanta, GA) with an isocratic 65:35 methanol:water mobile phase (1.0 mL/min). A 40 μL injection volume was used.

Fluorescence excitation and emission spectra of the porphyrin-embedded materials were collected in 96-well plate format with a Tecan XSafire monochromator-based microplate reader (1 nm resolution). Emission and excitation illumination wavelengths were selected to be those providing maximum fluorescence intensities for the given porphyrin. Fluorescence spectra of the porphyrin-functionalized materials (2 mg) were collected in a total volume of 20 μL (target + methanol). Controls were exposed to methanol only. Absorbance spectra of porphyrins in solution (95% methanol/5% water) were also collected using the plate reader (370 nm–770 nm at 1 nm resolution). Difference spectra are calculated as the point-by-point subtraction of the pre-exposure spectrum from the post-exposure spectrum. Peak fitting of the difference spectra where peak and trough positions resulted in overlapping curves was accomplished using Grams/AI v 8.0 (Thermo Scientific). The concentration dependence data presented is based on the average of measurements conducted in triplicate. Fitting of the concentration dependence data was accomplished in PSI-Plot v 8.51 (Poly Software International, Inc.).

For collection of reflectance spectra by scanner, porphyrin-embedded materials (5 mg) were placed into shell vials. Vials were placed on the surface of a Canon CanoScan N670U flatbed scanner with a control sample for color profile verification. Images of dry and wetted samples were collected. Vials for all wetted samples contained a total of 25 μL of solvent (target plus methanol). The scanned images were analyzed using Adobe PhotoShop CS3 Extended (version 10.0.1). Average RGB (red, green, blue) color values across each sample are reported here.

The paper by Smits [32] outlines a method to generate the seven reference spectra: white, red, blue, green, cyan, magenta, and yellow and a method to use these spectra to generate a reflectance spectrum for a given value of the RGB values. The algorithm uses weighted piecewise constant basis functions to define the spectrum. This reflectance spectrum is not a unique solution as many spectra (or metamers) can produce the same color. The focus of this methodology is to produce physically plausible spectra. A linear transform from XYZ to RGB space is defined from 360–800 nm in 5 nm increments, and the spectra are solved for as solutions to the constraints required to produce physically plausible spectra (Supporting Information, Figure S10). Smits makes freely available for download a Matlab script that can be used to generate the required reference spectra using IUT-R BT.709 standards to define the color space [39]. These functions determined the highest resolution that could be obtained for the reference spectra. Once the reference spectra are obtained a straight forward set of rules are applied to convert any RGB set into a simulated spectra.

## 4. Conclusions

We have described the design of a construct for the optical detection of hydrocarbon targets based on a porphyrin-embedded porous organosilicate material. The combination of the porphyrin and the organosilicate material makes it possible to utilize the characteristics of both leading to enhancement in detection capabilities. Here, the binding of target by the organosilicate leads to a locally high concentration of target in the vicinity of the porphyrin indicator leading to a strong change in spectrophotometric characteristics. The effective binding affinity of the construct for the target is also higher than that observed for the same porphyrin in solution.

The failure of C_4_TPP variants indicates the need for careful consideration of the immobilization techniques used. The goal in immobilizing the porphyrin onto the pore surface is to put the porphyrin in close proximity to the target concentrating sites while leaving it free to interact with targets in a solution-like manner. The dramatic change in the interaction characteristics indicates that the porphyrins are not in a solution-like environment, *i.e.*, there is likely an interaction between the porphyrin and the pore wall. This interaction can change the π-bond conformation of the porphyrin, leading to a change in interaction characteristics, or it can prevent the interaction of the porphyrin with the target. The effect could be considered similar to the effect of porphyrin aggregation. Changing the orientation of the porphyrin with respect to the pore wall either by reducing the number of bonds between the porphyrin and the amine functional groups or by crowding the porphyrin during immobilization could reduce the porphyrin surface interactions and provide a more solution-like porphyrin-target interaction. Varying the ratio of EDC reagent to porphyrin or that of the porphyrin to amine surface groups, for example, could produce a C_4_TPP construct with improved spectrophotometric characteristics.

The utilization of these constructs in the reflectance-based detection technique described previously [2] has been demonstrated. Early efforts in this area focused on indicators in thin films or as spotted arrays. Aggregation of the indicators in those formats as well as diffusion limitations resulting from the thin films limited the applicability of the technique. A more recent effort demonstrated the potential of porphyrin-embedded organosilicate materials and introduced the idea of RGB value utilization [4]. During the preparation of this manuscript, another report was published demonstrating the use of an array of similar porous indicators [8]. Modifications including the methyl, phenethyl, and octyl functionalities evaluated here were included in that study. The porous constructs of the recent report and those described here offer higher concentrations of indicators without the aggregation issues presented by deposition onto solid surfaces. The reduction in diffusion considerations also provides the potential for use of this technique in real-time monitoring as opposed to the cumulative dosimetry-type monitoring applications for which the thin films were well suited.

## Supporting Information



## Figures and Tables

**Table 1.** Material Characteristics.

**Material**		**Surface Area (m^2^/g)**	**Pore Volume (cm^3^/g)**	**Pore Diameter (Å)**	Benzene Bound * (mg/g)	**Benzene Bound ^§^ (mg/g)**
Biphenyl-bridged material	BP2	831	1.22	51	48	200
Benzene-bridged material	B100	1,132	0.98	35	118	200
Diethylbenzene-bridged material	DEB	468	0.48	49	72	197
Material incorporating methyl groups	TM1	803	1.11	89	119	180
Material with terminal phenyl groups	Ph1	1,044	0.58	22	64	91
Material with terminal phenethyl groups	PhE1	522	0.31	<20	37	50
Material with terminal octyl groups	OTS	344	0.80	228 & 626 ^+^	64	94

+ Broad distribution of pores, see Figure 3B.

* Bound after 1 hr from headspace experiment using 53 mg benzene and 200 mg sorbent.

§ Bound after 1 hr following application of liquid benzene (53 mg) to sorbent (200 mg).

**Table 2.** Interaction of porphyrins with targets in solution. Δλ and ΔI calculated based on absorbance changes for porphyrins (4 μM) in the absence/presence of targets (200 mM, in 95% methanol) using the rapid screening protocol. Association constants and extinction coefficient changes calculated from binding isotherms generated for porphyrins (3.3 μM) exposed to targets in methanol. Data for additional metalloporphyrins is provided in Supporting Information, Tables S2 and S3.

**Porphyrin**	**Target**	**Δλ**	**ΔI**	**K_11_ (1/M)**	**Δ′_11_ Peak (A/M)**	**Δ′_11_ Trough (A/M)**
FeC_1_TPP	Benzene	8	0.502	1.0	3,500	2,540
	Toluene	−	−	−	−	−
	Hexane	8	0.462	2.5	5,240	1,340
C_4_TPP	Benzene	9	0.078	2.0	65,910	42,770
	Toluene	11	0.215	0.9	76,130	43,500
	Hexane	8	0.039	1.7	107,600	32,550
MnC_4_TPP	Benzene	−	0.188	1.0	−	132,400
	Toluene	−	0.127	7.1	−	38,060
	Hexane	−	0.083	7.3	−	22,720

**Table 3.** Interaction of porphyrin embedded materials with benzene, toluene, and hexane. Peak and trough positions are taken from difference spectra.

**Porphyrin**	**Target**	**Excitation**		**Emission**	
		**Peak (nm)**	**Trough (nm)**	**Peak (nm)**	**Trough (nm)**
C_1_TPP	Benzene	426	418	662	642
	Toluene	428	413	659	644
	Hexane	422	414	−	646
FeC_4_TPP	Benzene	428	412	663	648
	Toluene	426	414	670	664
	Hexane	430	412	654	718
MnC_1_TPP	Benzene	430	418	652	−
	Toluene	−	−	−	650
	Hexane	432	412	−	656
