# Supplementary material for: Porphyrin-Embedded Silicate Materials for Detection of Hydrocarbon Solvents
Source: Sensors (Basel). 2011 Jan 14;11(1):886–904. doi: 10.3390/s110100886 (PMC3274106; doi:10.3390/s110100886)

*Sensors* **2011**, *11*, 886-904; doi:10.3390/s110100886

OPEN ACCESS

**sensors**

**ISSN 1424-8220**

www.mdpi.com/journal/sensors

*Supporting Information*

# Porphyrin-Embedded Silicate Materials for Detection of Hydrocarbon Solvents

**Brandy J. Johnson *, Nicole E. Anderson, Paul T. Charles, Anthony P. Malanoski, Brian J. Melde, Mansoor Nasir and Jeffrey R. Deschamps**

Center for Bio/Molecular Science and Engineering, Naval Research Laboratory, Washington, D.C. 20375, USA; E-Mails: nanders2@umd.edu (N.E.A); paul.charles@nrl.navy.mil (P.T.C.); anthony.malanoski@nrl.navy.mil (A.P.M.); brian.melde@nrl.navy.mil (B.J.M.); mansoor.nasir@nrl.navy.mil (M.N.); jeff.deschamps@nrl.navy.mil (J.R.D.)

* Author to whom correspondence should be addressed; E-Mail: brandy.white@nrl.navy.mil; Tel.: +1-202-404-6100; Fax: +1-202-767-9598.

*Received: 1 December 2010; in revised form: 5 January 2011 / Accepted: 13 January 2011 / Published: 14 January 2011*

---

The supplementary data supplied here provides complete results of structural characterization and image analysis for exposure of the various materials to hexane, benzene, and toluene. A list of the metal salts used to generate metalloporphyrin variants is provided. Additional absorbance and fluorescence results and reflectance spectra are also included.

---

**Table S1.** Metal salts used in the preparation of metalloporphyrin variants.

| | | |
|---|---|---|
| magnesium chloride | copper (II) chloride | vanadium (III) bromide |
| yttrium (III) chloride | zinc chloride | ruthenium (III) chloride |
| titanium (II) chloride | silver chloride | praseodynium (III) chloride |
| manganese (II) chloride | cadmium chloride | cerium (III) chloride |
| iron (III) chloride | tin (II) chloride | europium (II) chloride |
| cobalt (II) chloride | platinum (IV) chloride | osmium (III) chloride |
| nickle (II) chloride | gold (III) chloride | |

**Table S2.** Results of rapid screening for interaction of porphyrins with targets in solution. For $C_1$TPP and $C_4$TPP, results for the four selected candidates and the next four highest performers are provided. For $C_1S_3$TPP, results for the top six performers are provided.

| Porphyrin | Target | Δ λ | Δ I | Porphyrin | Target | Δ λ | Δ I |
|---|---|---|---|---|---|---|---|
| $C_1$TPP | Benzene | 8 | 0.062 | $C_4$TPP | Benzene | 9 | 0.078 |
| | Toluene | 9 | 0.134 | | Toluene | 11 | 0.215 |
| | Hexane | -- | -- | | Hexane | 8 | 0.039 |
| $FeC_1$TPP | Benzene | 8 | 0.502 | $FeC_4$TPP | Benzene | -- | 0.038 |
| | Toluene | -- | -- | | Toluene | -- | 0.094 |
| | Hexane | 8 | 0.462 | | Hexane | 10 | 0.096 |
| $CuC_1$TPP | Benzene | 10 | 0.145 | $MnC_4$TPP | Benzene | -- | 0.188 |
| | Toluene | 12 | 0.222 | | Toluene | -- | 0.127 |
| | Hexane | -- | -- | | Hexane | -- | 0.083 |
| $MnC_1$TPP | Benzene | 10 | 0.139 | $ZnC_4$TPP | Benzene | 9 | 0.073 |
| | Toluene | 8 | 0.199 | | Toluene | 8 | 0.054 |
| | Hexane | 7 | 0.030 | | Hexane | -- | 0.095 |
| $ZnC_1$TPP | Benzene | 6 | 0.083 | $CoC_4$TPP | Benzene | 9 | 0.042 |
| | Toluene | 7 | 0.053 | | Toluene | 8 | 0.043 |
| | Hexane | 4 | 0.016 | | Hexane | 10 | 0.018 |
| $CoC_1$TPP | Benzene | 8 | 0.097 | $CuC_4$TPP | Benzene | 9 | 0.076 |
| | Toluene | 9 | 0.033 | | Toluene | 7 | 0.062 |
| | Hexane | 8 | 0.016 | | Hexane | 7 | 0.014 |
| $MgC_1$TPP | Benzene | 9 | 0.098 | $MgC_4$TPP | Benzene | 8 | 0.058 |
| | Toluene | 8 | 0.084 | | Toluene | 8 | 0.039 |
| | Hexane | 8 | 0.023 | | Hexane | -- | 0.043 |
| $NiC_1$TPP | Benzene | 9 | 0.093 | $NiC_4$TPP | Benzene | 10 | 0.047 |
| | Toluene | 10 | 0.049 | | Toluene | 9 | 0.084 |
| | Hexane | 12 | 0.056 | | Hexane | -- | 0.026 |
| $C_1S_3$TPP | Benzene | 6 | 0.015 | $MnC_1S_3$TPP | Benzene | 9 | 0.012 |
| | Toluene | 6 | 0.032 | | Toluene | 6 | 0.009 |
| | Hexane | 6 | 0.01 | | Hexane | 8 | 0.033 |
| $FeC_1S_3$TPP | Benzene | -- | 0.018 | $CuC_1S_3$TPP | Benzene | -- | 0.023 |
| | Toluene | -- | 0.011 | | Toluene | -- | 0.024 |
| | Hexane | -- | -- | | Hexane | 6 | 0.02 |
| $NiC_1S_3$TPP | Benzene | -- | 0.016 | $ZnC_1S_3$TPP | Benzene | 8 | 0.029 |
| | Toluene | -- | 0.014 | | Toluene | 6 | 0.008 |
| | Hexane | -- | -- | | Hexane | -- | 0.004 |

**Table S3.** Interaction of candidate porphyrins with targets in solution.

| | | Rapid Screening | | Binding Isotherm | | |
|---|---|---|---|---|---|---|
| **Porphyrin** | **Target** | **$\Delta\ \lambda$** | **$\Delta$ I** | **$K_{11}$ (1/M)** | **$\Delta\varepsilon_{11}$ Peak (A/M)** | **$\Delta\varepsilon_{11}$ Trough (A/M)** |
| $C_1$TPP | Benzene | 8 | 0.062 | 2.1 | 1,250 | 2,580 |
| | Toluene | 9 | 0.134 | 6.3 | 522 | 586 |
| | Hexane | -- | -- | -- | -- | -- |
| $FeC_1$TPP | Benzene | 8 | 0.502 | 1.0 | 3,500 | 2,540 |
| | Toluene | -- | -- | -- | -- | -- |
| | Hexane | 8 | 0.462 | 2.5 | 5,240 | 1,340 |
| $CuC_1$TPP | Benzene | 10 | 0.145 | 150 | 20,860 | 398 |
| | Toluene | 12 | 0.222 | 9.8 | 594 | 1,440 |
| | Hexane | -- | -- | -- | -- | -- |
| $MnC_1$TPP | Benzene | 10 | 0.139 | 10 | 19,030 | 18,680 |
| | Toluene | 8 | 0.199 | 5.1 | 178,000 | 12,180 |
| | Hexane | 7 | 0.030 | 120 | 3,210 | 2,650 |
| $C_4$TPP | Benzene | 9 | 0.078 | 2.0 | 65,910 | 42,770 |
| | Toluene | 11 | 0.215 | 0.9 | 76,130 | 43,500 |
| | Hexane | 8 | 0.039 | 1.7 | 107,600 | 32,550 |
| $FeC_4$TPP | Benzene | -- | 0.038 | 17 | -- | 53,420 |
| | Toluene | -- | 0.094 | 26 | -- | 25,380 |
| | Hexane | 10 | 0.096 | 13 | -- | 30,450 |
| $MnC_4$TPP | Benzene | -- | 0.188 | 1.0 | -- | 132,400 |
| | Toluene | -- | 0.127 | 7.1 | -- | 38,060 |
| | Hexane | -- | 0.083 | 7.3 | -- | 22,720 |
| $ZnC_4$TPP | Benzene | 9 | 0.073 | 0.7 | 60,900 | 43,500 |
| | Toluene | 8 | 0.054 | 5.5 | 27,680 | 12,690 |
| | Hexane | -- | 0.095 | 8.0 | -- | 19,030 |

**Figure S1.** Structural characterization. Panel A, nitrogen sorption isotherms (DEB shifted by +240 and TM1 shifted by +250). Panel B, pore size distributions. Panel C, XRD spectra. (DEB–blue, TM1–red, Ph1–black, PhE1–green).

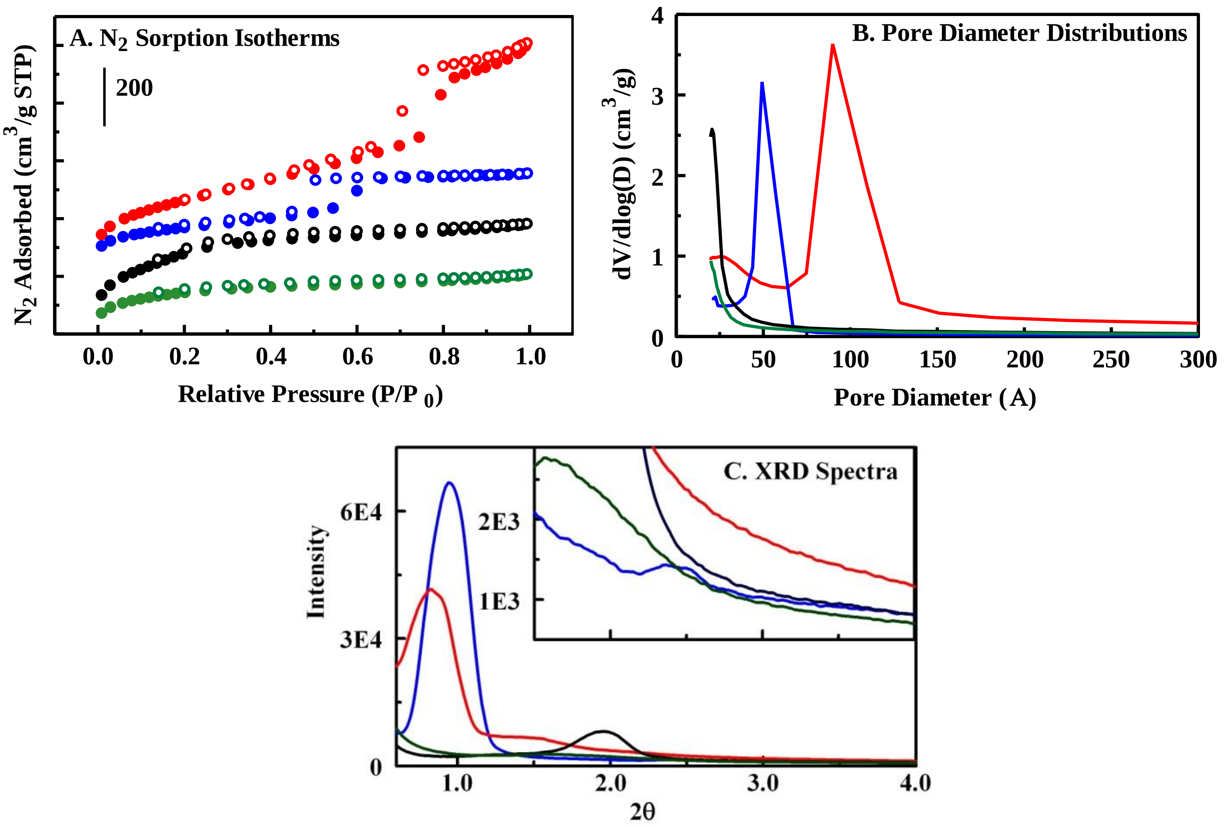


**Figure S2.** BTEX binding capacity. Shown here is the amount of each BTEX component bound by the sorbents (200 mg). BTEX (209 mg—equal volumes benzene, toluene, ethylbenzene, and xylene) binding was evaluated in vapor phase.

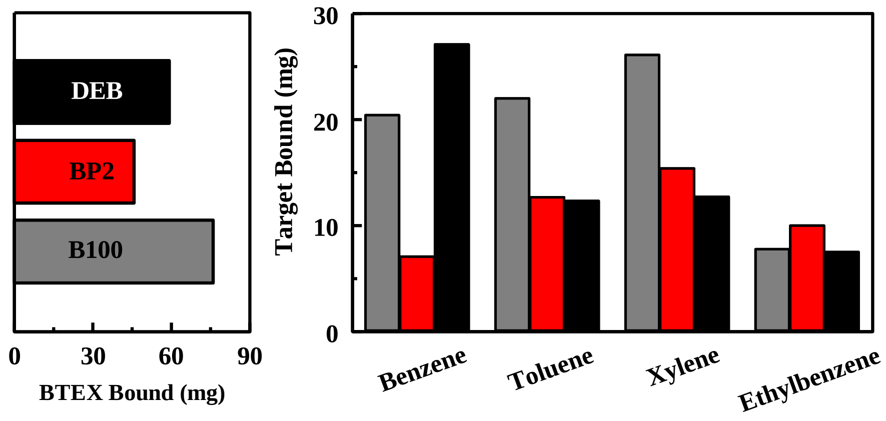

**Figure S3.** Dependence on concentration of the interaction between metalloporphyrins and targets in solution (benzene–red, toluene–green, hexane–blue).

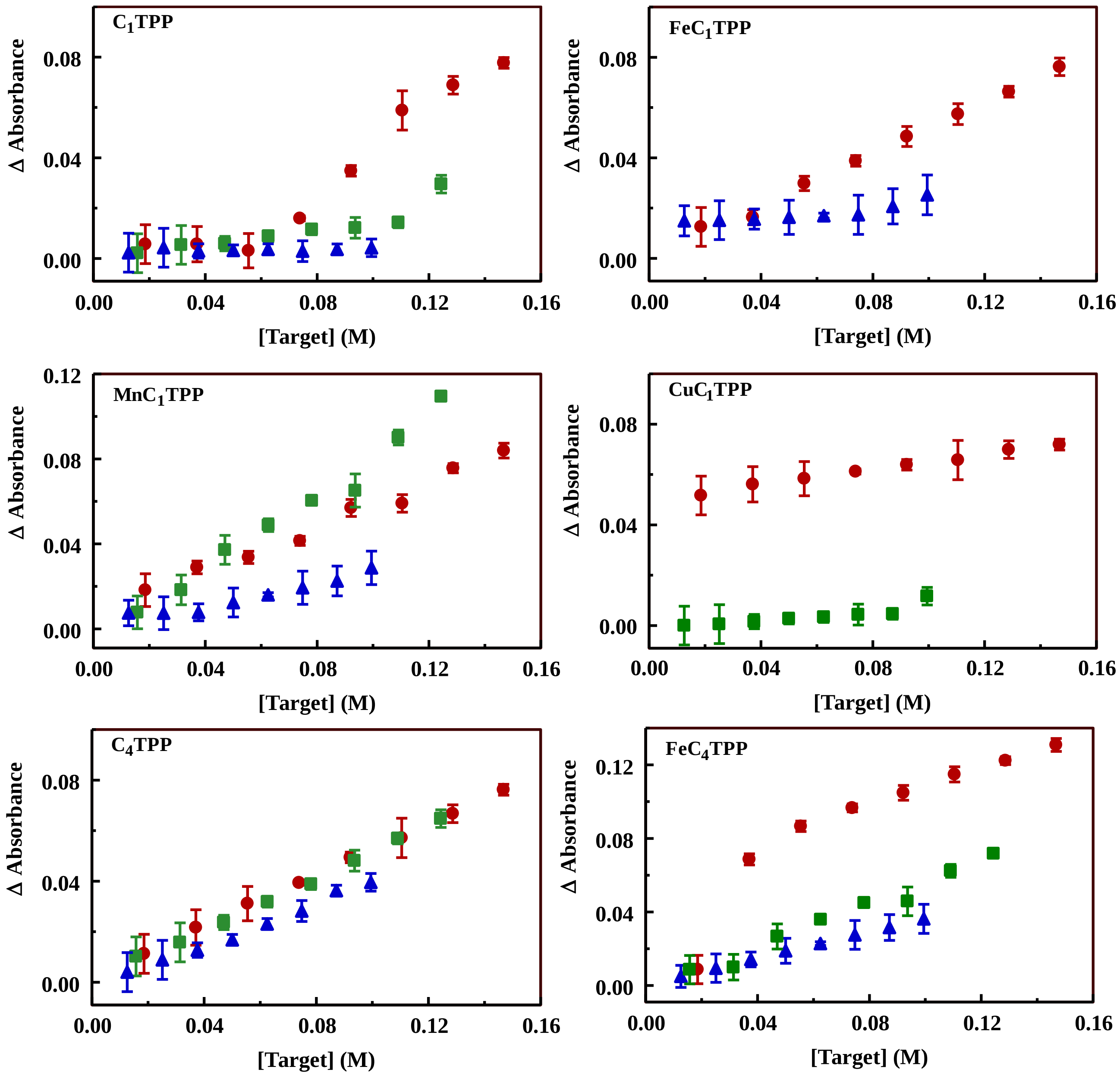

**Figure S4.** Interaction of $FeC_1TPP$-embedded B100 with targets. Shown here are the changes in fluorescence characteristics and the dependence of RGB image color values on target concentration for the interaction of targets with $FeC_1TPP$-embedded B100. Changes in fluorescence intensity versus concentration are based on peak/trough differences generated by peak fitting the excitation difference spectra.

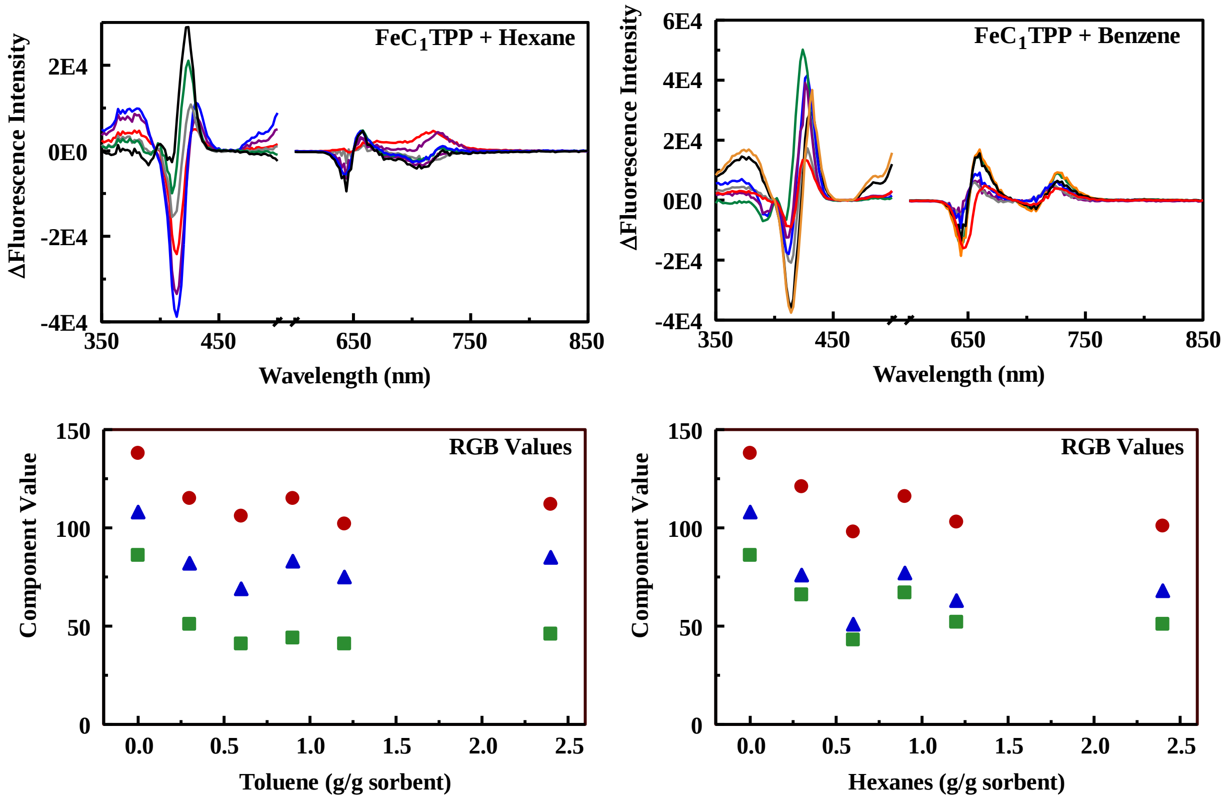

**Figure S5.** Interaction of $C_1$TPP-embedded B100 with targets. Shown here are the changes in fluorescence characteristics, simulated images generated based on average RGB values, and the dependence of RGB image color values on target concentration for the interaction of targets with $C_1$TPP-embedded B100. Changes in fluorescence intensity versus concentration are based on peak/trough differences generated by peak fitting the excitation difference spectra.

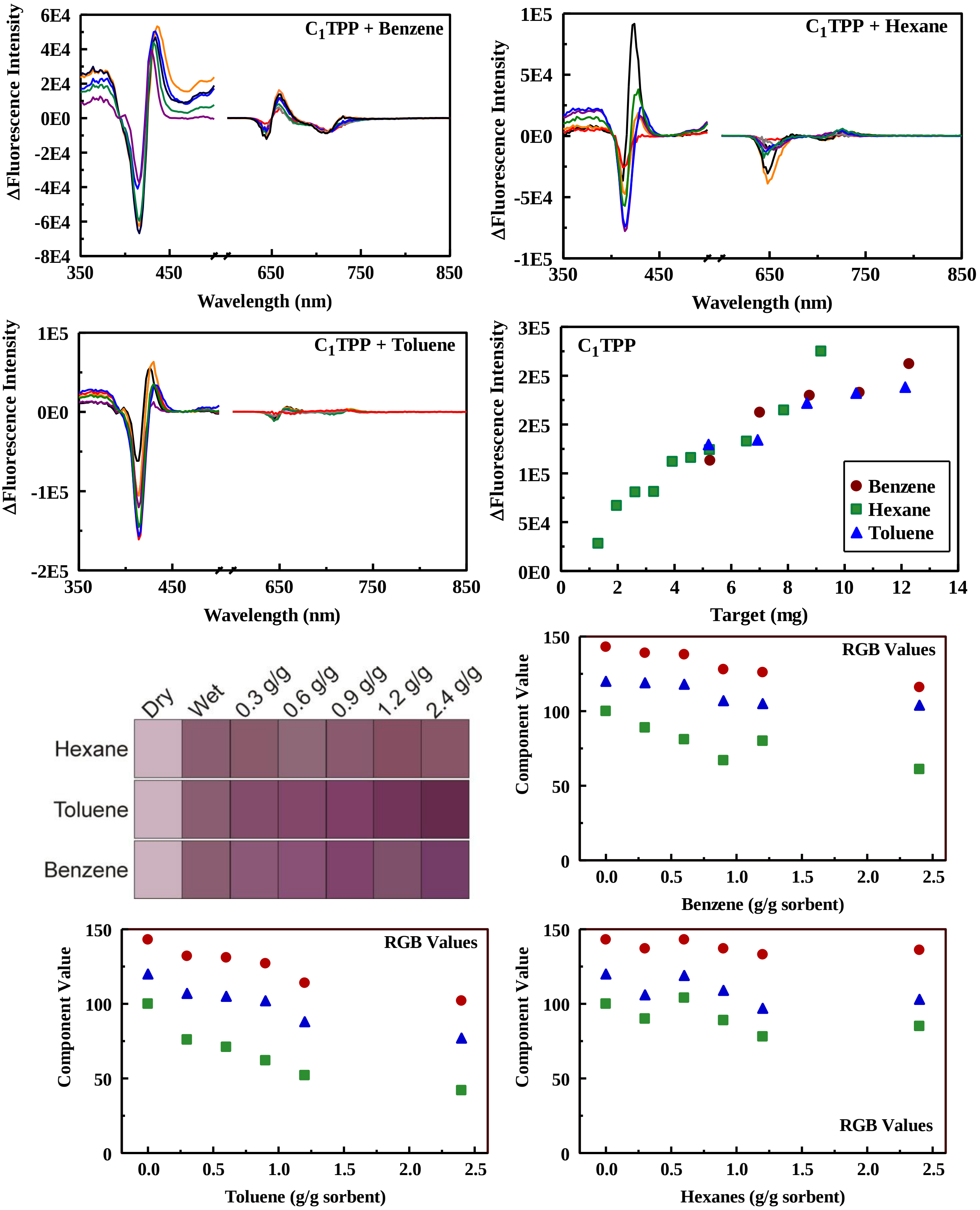

**Figure S6.** Interaction of $MnC_1TPP$-embedded B100 with targets. Shown here are the changes in fluorescence characteristics, simulated images generated based on average RGB values, and the dependence of RGB image color values on target concentration for the interaction of targets with $MnC_1TPP$-embedded B100. Changes in fluorescence intensity versus concentration are based on peak/trough differences generated by peak fitting the excitation difference spectra.

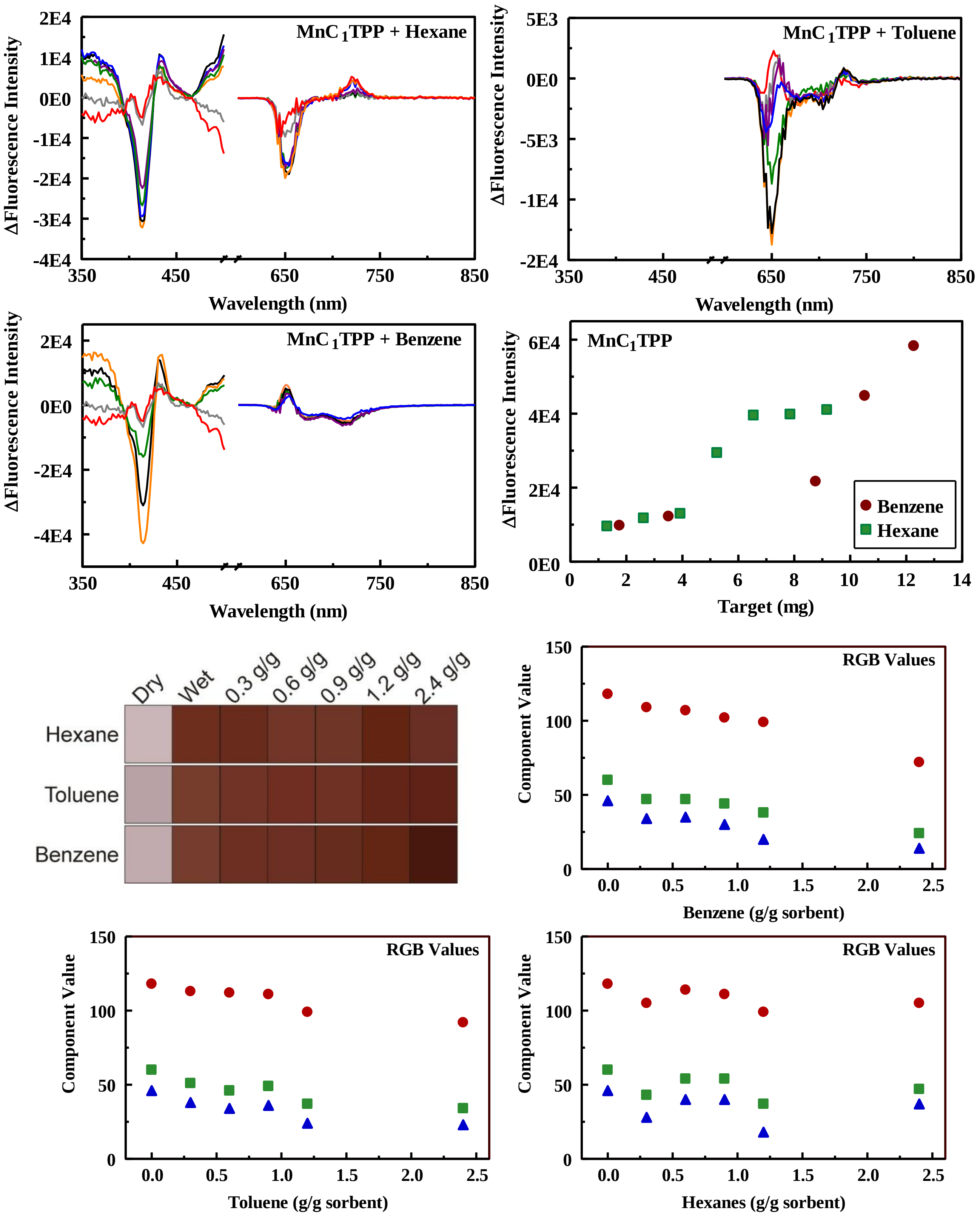

**Figure S7.** Interaction of $C_4$TPP-embedded B100 with targets. Shown here are the changes in fluorescence characteristics, simulated images generated based on average RGB values, and the dependence of RGB image color values on target concentration for the interaction of targets with $C_4$TPP-embedded B100. Changes in fluorescence intensity versus concentration are based on peak/trough differences generated based on the emission difference spectra.

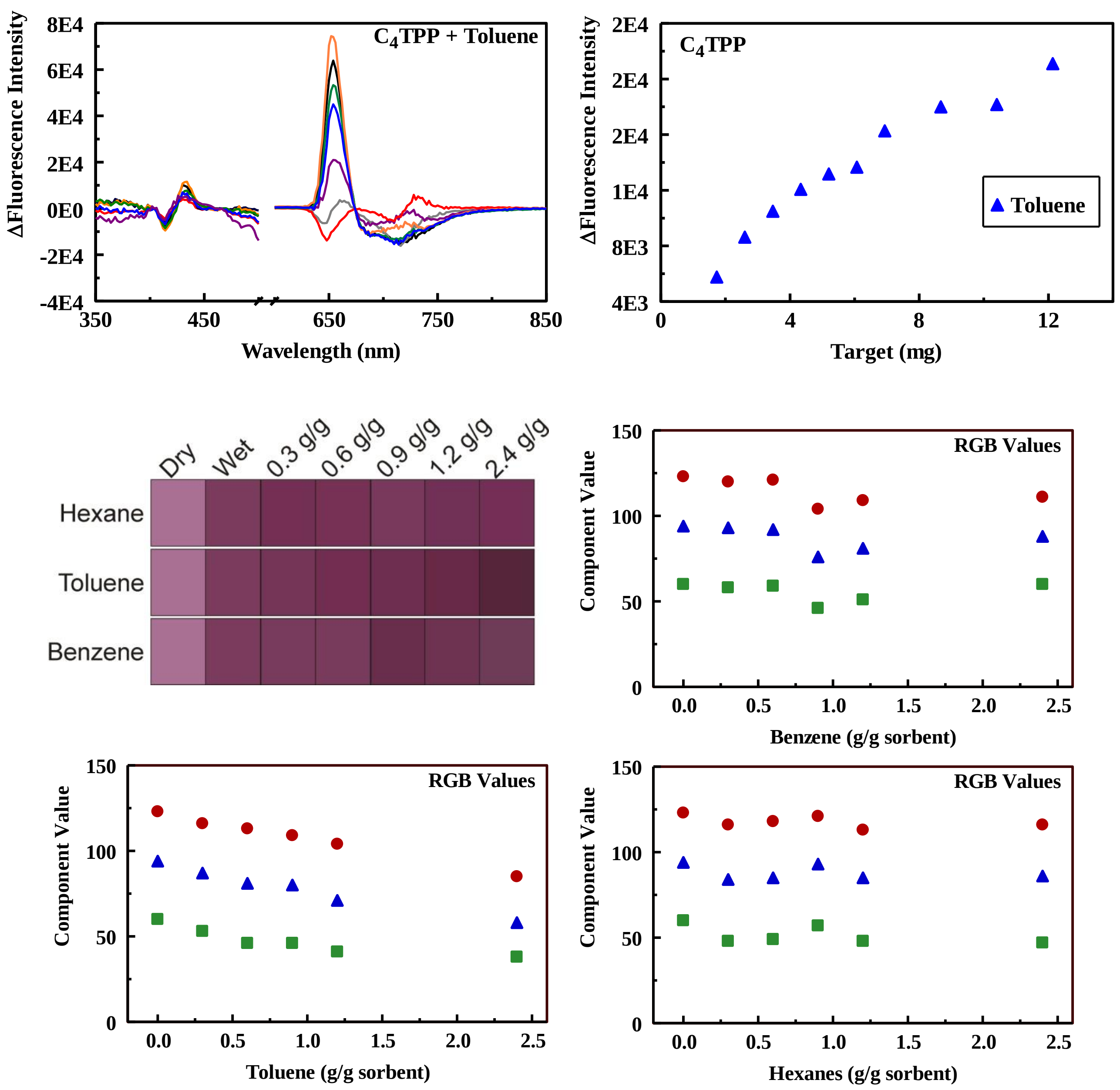

**Figure S8.** Interaction of $FeC_4TPP$-embedded B100 with targets. Shown here are the changes in fluorescence characteristics, simulated images generated based on average RGB values, and the dependence of RGB image color values on target concentration for the interaction of targets with $FeC_4TPP$-embedded B100. Changes in fluorescence intensity versus concentration are based on peak/trough differences from emission difference spectra.

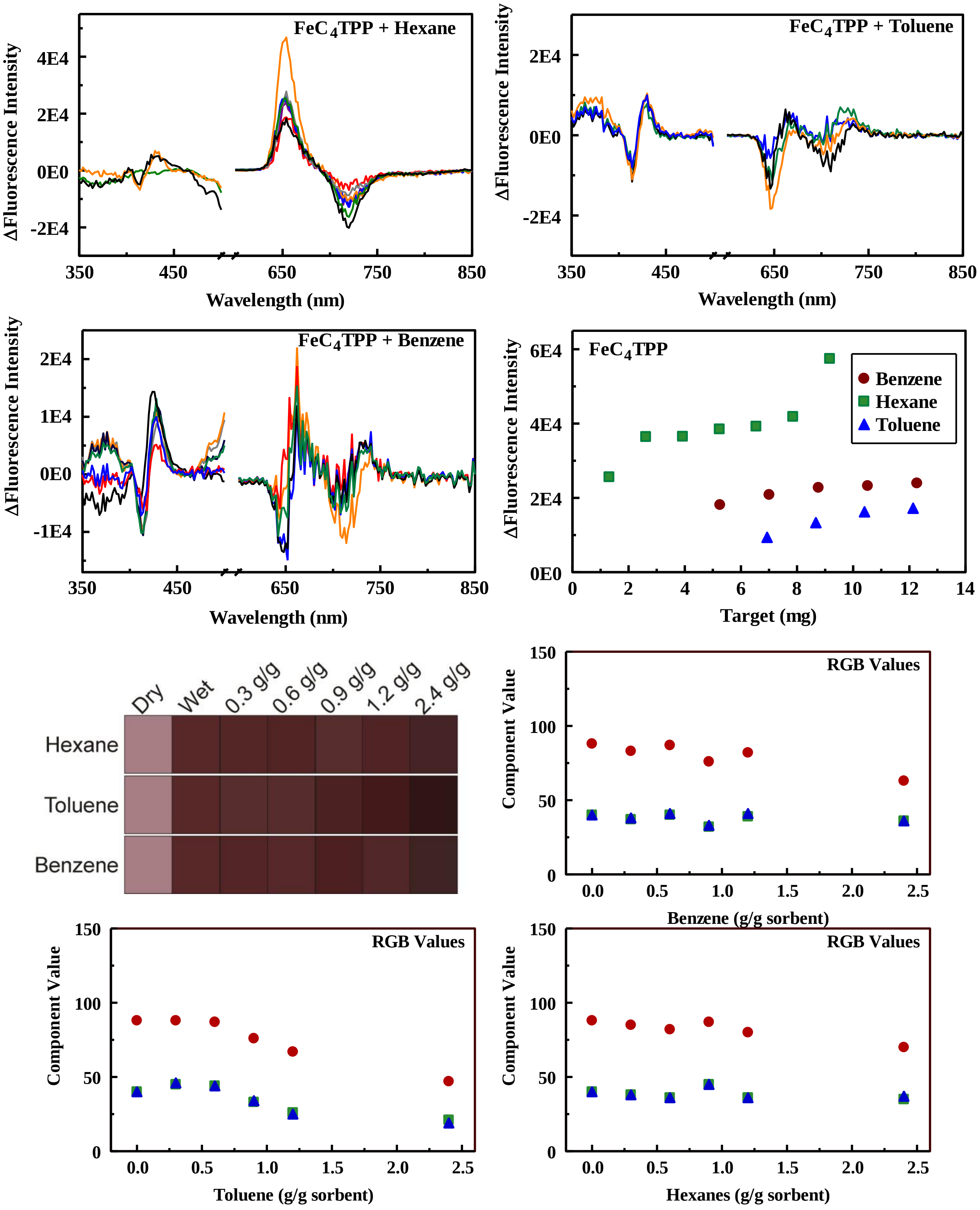

**Figure S9.** Interaction of $MnC_4TPP$-embedded B100 with targets. Shown here are simulated images generated based on average RGB values for $FeC_1TPP$-embedded B100 following exposure to varying target concentrations and the dependence of RGB image color values on target concentration for the interaction of the targets with $MnC_4TPP$-embedded B100. No changes in fluorescence were observed upon exposure of $MnC_4TPP$-embedded B100 to the targets.

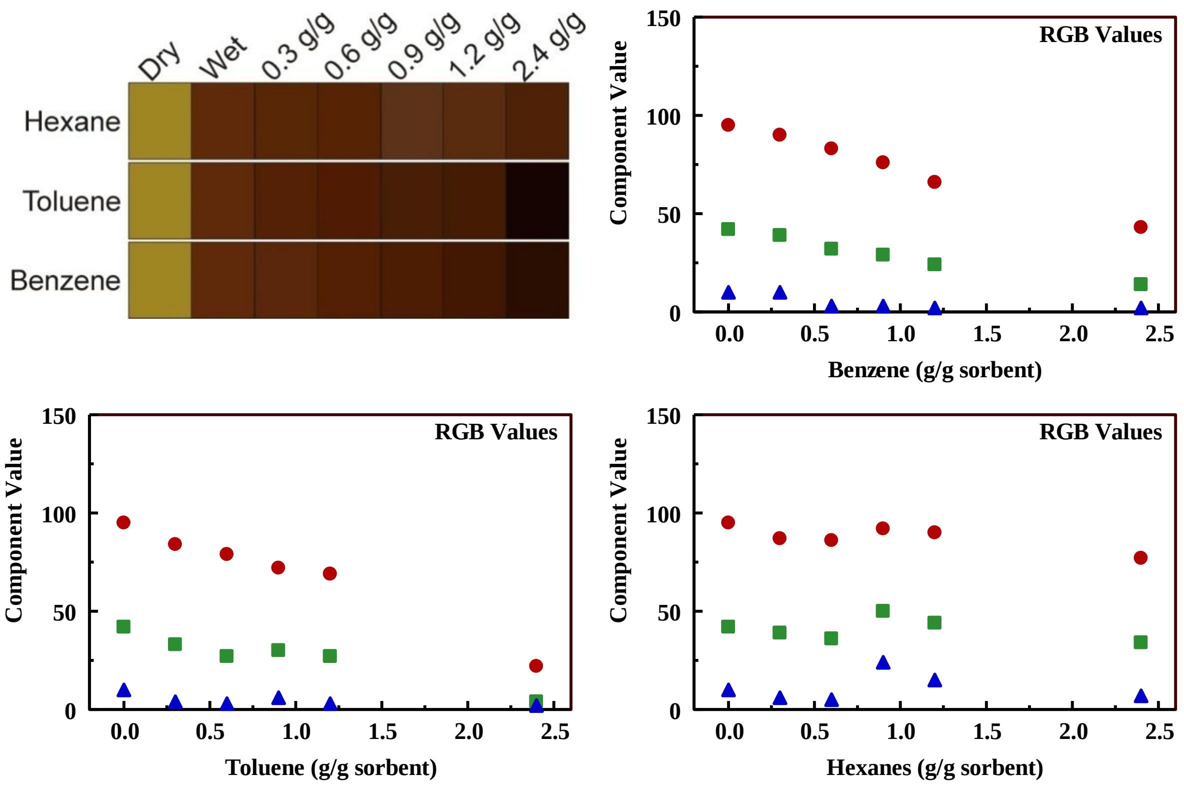

**Figure S10.** Reflectance spectra. Spectra for the seven different colors used for conversion from RGB to reflectance values. For the reason of solution convergence, the algorithm ignores scaling issues when converting from spectra to RGB leading to some reflectance values greater than 1. A penalty factor is added to the cost function whenever any value of the spectrum exceeds 1.

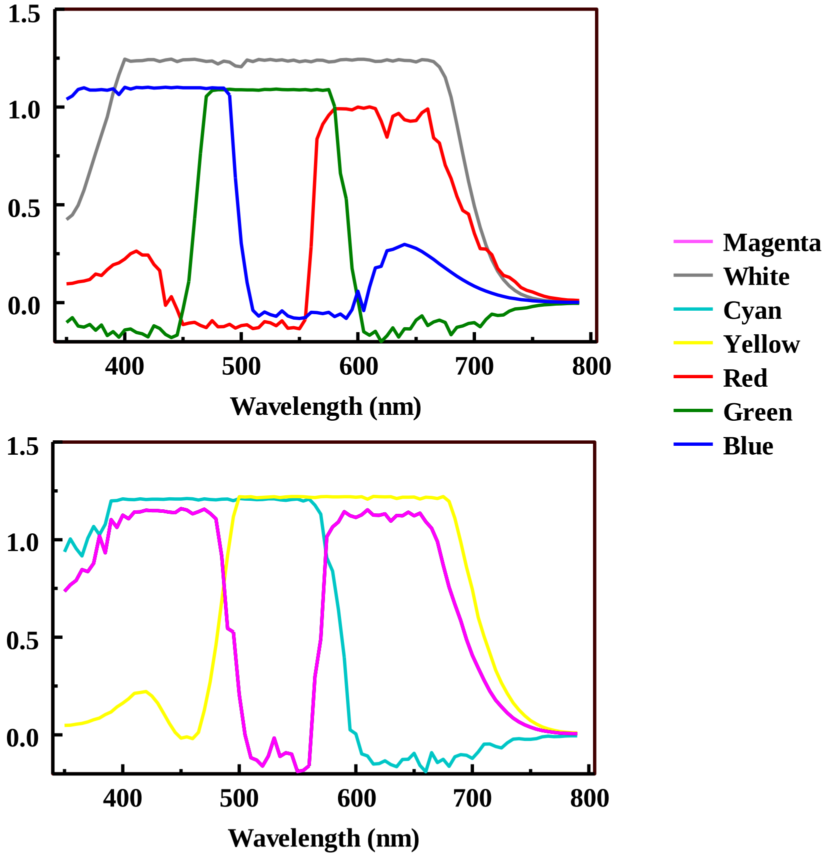

**Figure S11.** Simulated spectra for interaction of $C_1$TPP-embedded B100 (5 mg) with the targets. Shown here are reflectance spectra generated based on RGB values (Figure S5) using the color spectra from Figure S10.

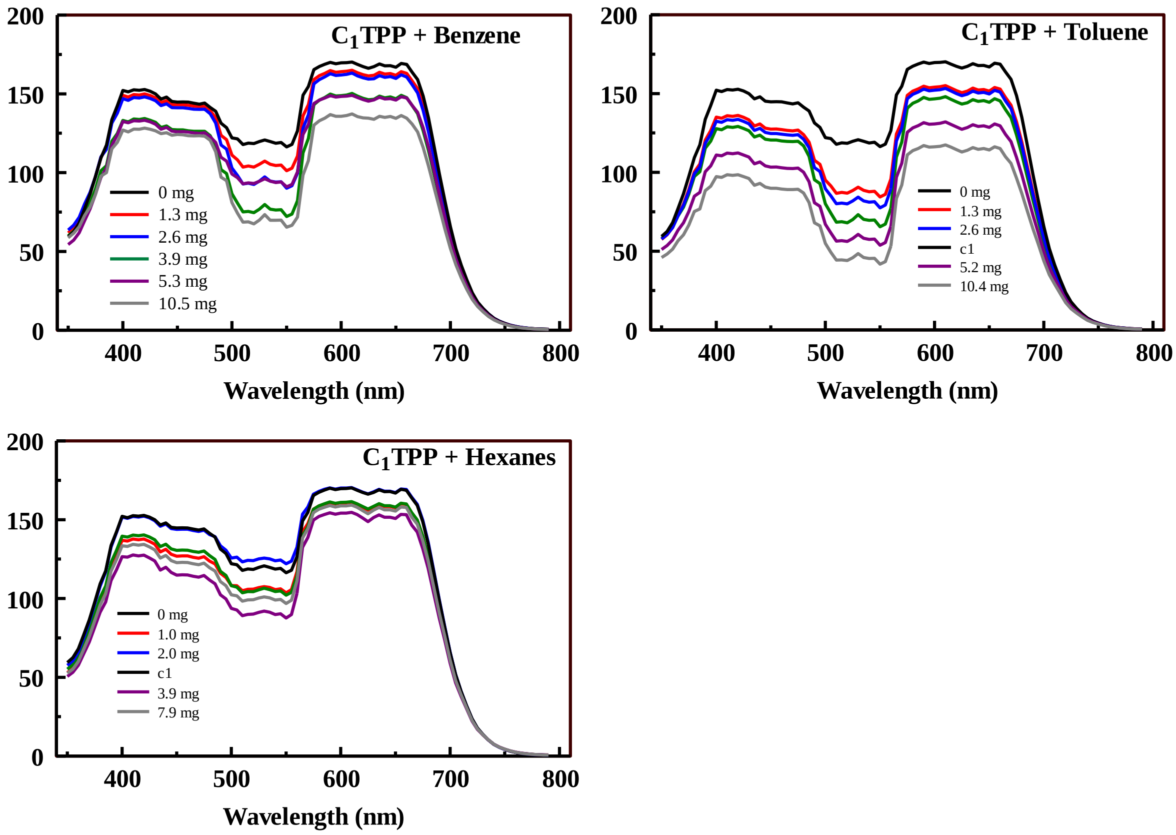


**Figure S12.** Simulated spectra for interaction of $FeC_1$TPP-embedded B100 (5 mg) with the targets. Shown here are reflectance spectra generated based on RGB values (Figure S4) using the color spectra from Figure S10.

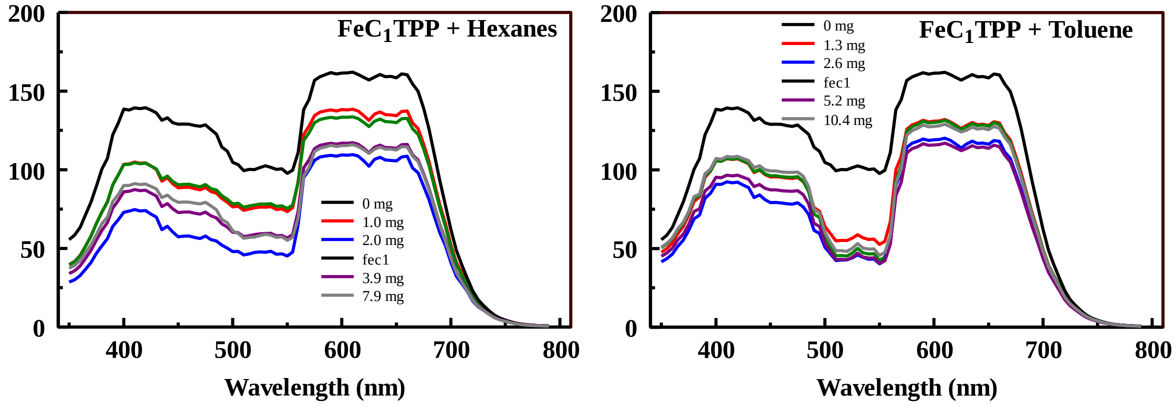

**Figure S13.** Simulated spectra for interaction of $MnC_1TPP$-embedded B100 (5 mg) with the targets. Shown here are reflectance spectra generated based on RGB values (Figure S6) using the color spectra from Figure S10.

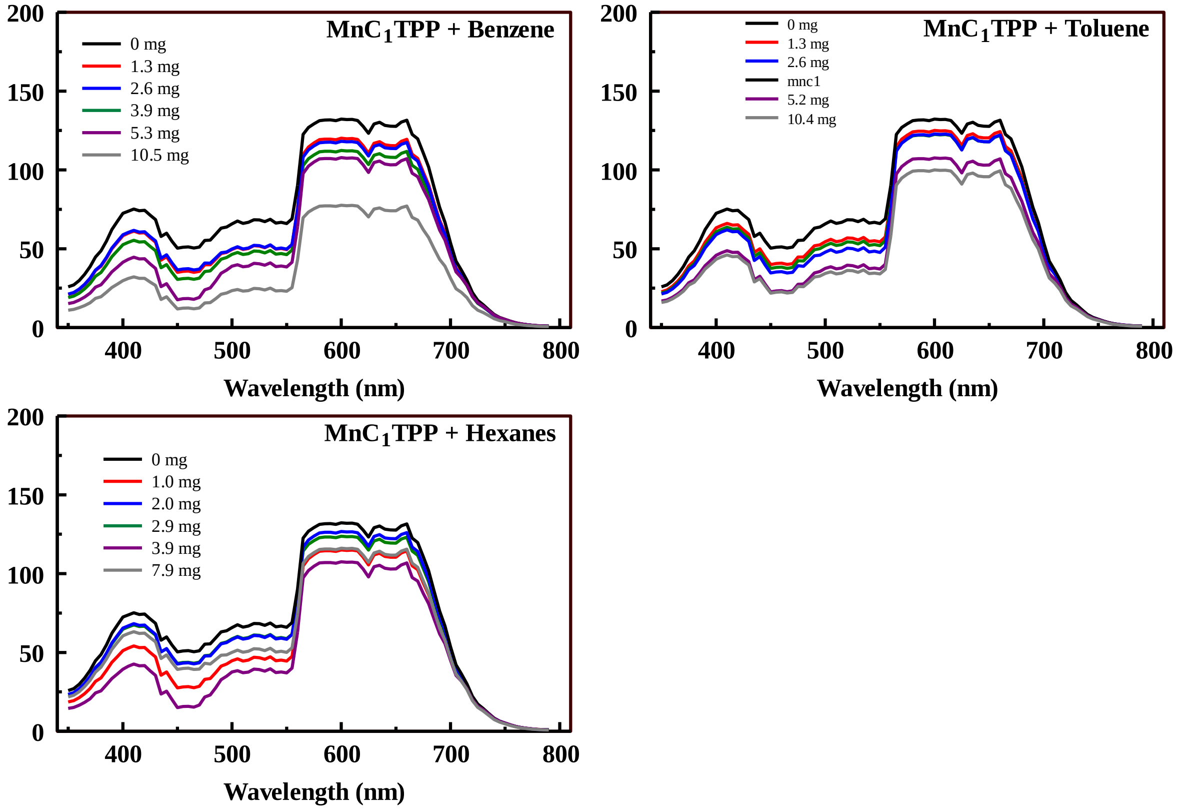

**Figure S14.** Simulated spectra for interaction of $C_4$TPP-embedded B100 (5 mg) with the targets. Shown here are reflectance spectra generated based on RGB values (Figure S7) using the color spectra from Figure S10.

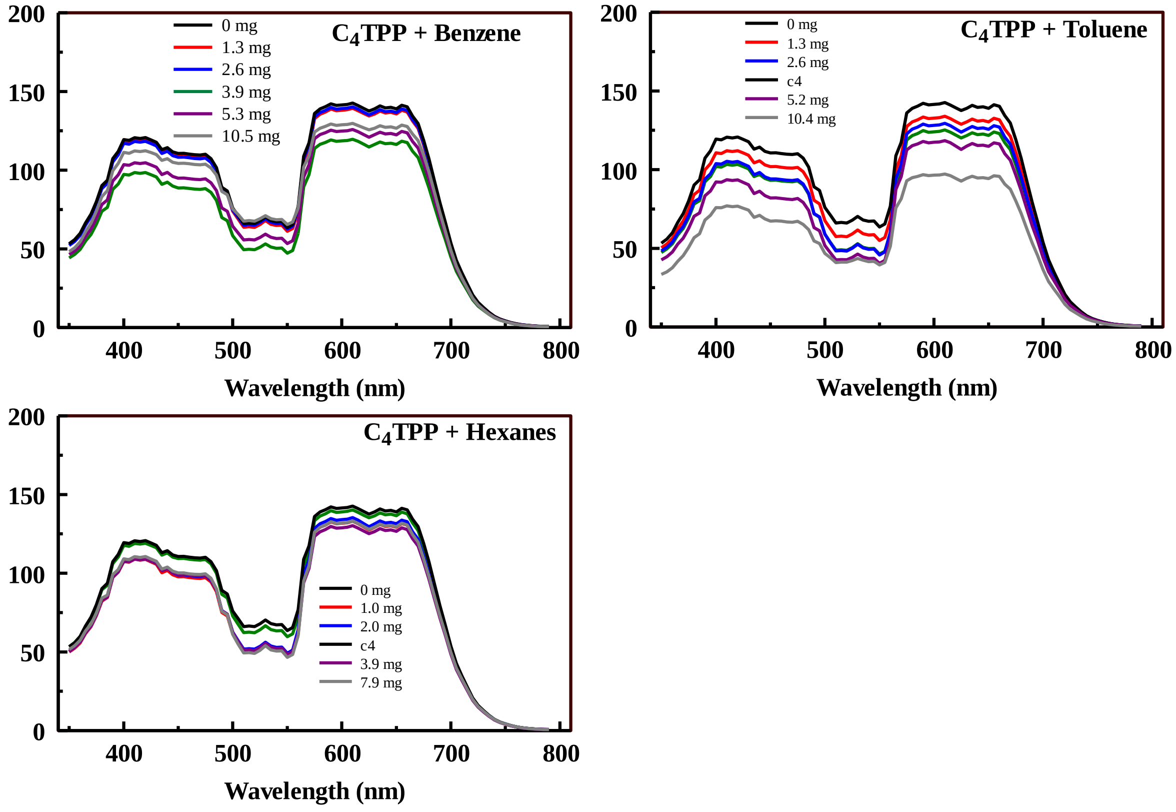

**Figure S15.** Simulated spectra for interaction of $FeC_4TPP$-embedded B100 (5 mg) with the targets. Shown here are reflectance spectra generated based on RGB values (Figure S8) using the color spectra from Figure S10.

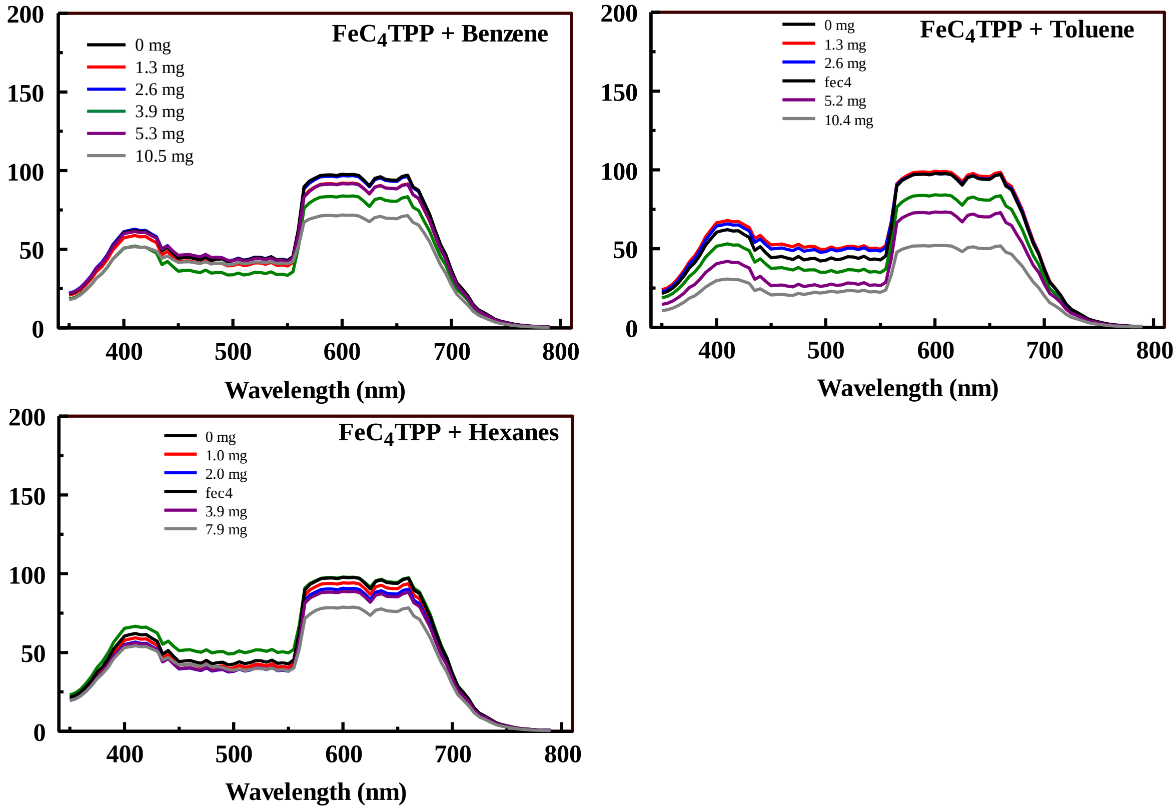

**Figure S16.** Simulated spectra for interaction of $MnC_4TPP$-embedded B100 (5 mg) with the targets. Shown here are reflectance spectra generated based on RGB values (Figure S9) using the color spectra from Figure S10.

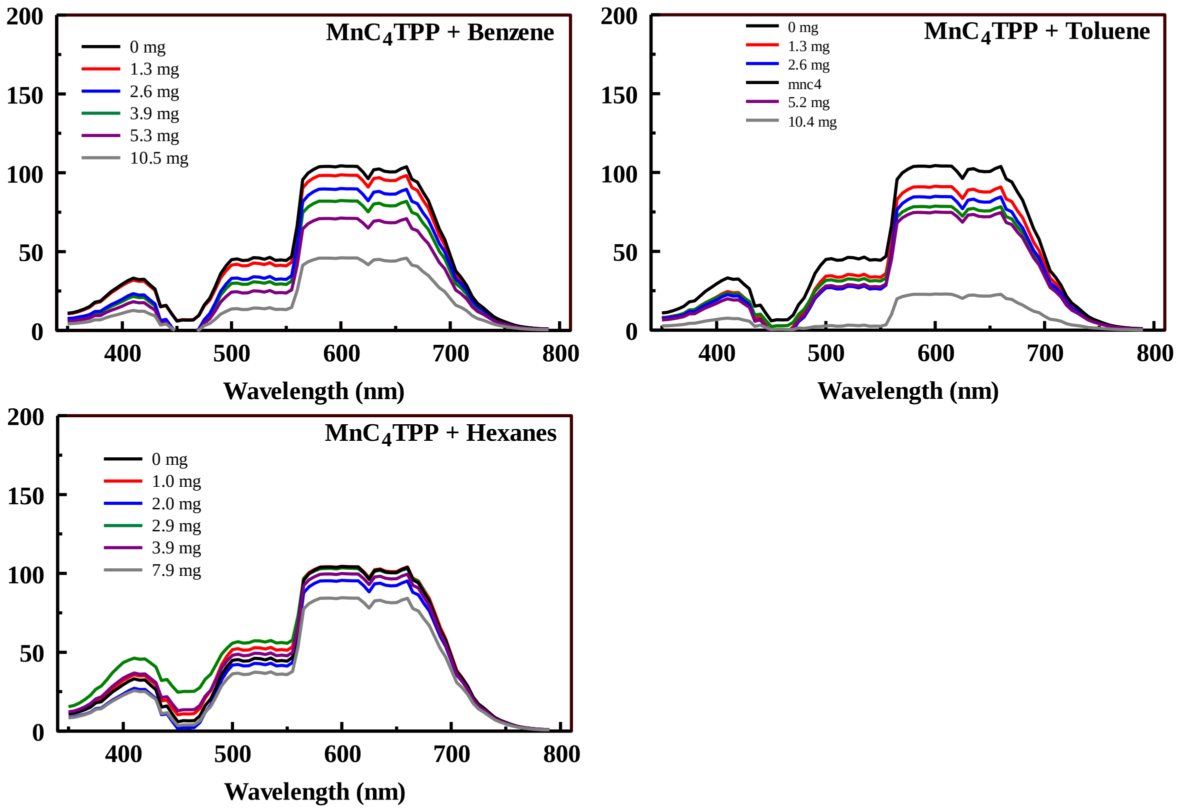

Supplement: Supplementary file 1 [file sensors-11-00886-s001.pdf]
